# A Chromosome-Level Genome Assembly of the Reef Stonefish (*Synanceia verrucosa*) Provides Novel Insights into Stonustoxin (*sntx*) Genes

**DOI:** 10.1093/molbev/msad215

**Published:** 2023-09-14

**Authors:** Tianle Tang, Yu Huang, Chao Peng, Yanling Liao, Yunyun Lv, Qiong Shi, Bingmiao Gao

**Affiliations:** Key Laboratory of Tropical Translational Medicine of Ministry of Education, School of Pharmacy, Hainan Medical University, Haikou, Hainan, China; Shenzhen Key Lab of Marine Genomics, Guangdong Provincial Key Lab of Molecular Breeding in Marine Economic Animals, BGI Academy of Marine Sciences, BGI Marine, Shenzhen, Guangdong, China; Key Laboratory of Breeding Biotechnology and Sustainable Aquaculture, Institute of Hydrobiology, Chinese Academy of Sciences, Wuhan, Hubei, China; Shenzhen Key Lab of Marine Genomics, Guangdong Provincial Key Lab of Molecular Breeding in Marine Economic Animals, BGI Academy of Marine Sciences, BGI Marine, Shenzhen, Guangdong, China; BGI-Marine Research Institute for Biomedical Technology, Shenzhen Huahong Marine Biomedicine Co. Ltd., Shenzhen, Guangdong, China; Key Laboratory of Tropical Translational Medicine of Ministry of Education, School of Pharmacy, Hainan Medical University, Haikou, Hainan, China; Key Laboratory of Sichuan Province for Fishes Conservation and Utilization in the Upper Reaches of the Yangtze River, College of Life Sciences, Neijiang Normal University, Neijiang, Sichuan, China; Shenzhen Key Lab of Marine Genomics, Guangdong Provincial Key Lab of Molecular Breeding in Marine Economic Animals, BGI Academy of Marine Sciences, BGI Marine, Shenzhen, Guangdong, China; BGI-Marine Research Institute for Biomedical Technology, Shenzhen Huahong Marine Biomedicine Co. Ltd., Shenzhen, Guangdong, China; Key Laboratory of Sichuan Province for Fishes Conservation and Utilization in the Upper Reaches of the Yangtze River, College of Life Sciences, Neijiang Normal University, Neijiang, Sichuan, China; Key Laboratory of Tropical Translational Medicine of Ministry of Education, School of Pharmacy, Hainan Medical University, Haikou, Hainan, China

**Keywords:** reef stonefish, genome assembly, stonustoxin, multiomics, expression, evolution

## Abstract

Reef stonefish (*Synanceia verrucosa*) is one of the most venomous fishes, but its biomedical study has been restricted to molecular cloning and purification of its toxins, instead of high-throughput genetic research on related toxin genes. In this study, we constructed a chromosome-level haplotypic genome assembly for the reef stonefish. The genome was assembled into 24 pseudo-chromosomes, and the length totaled 689.74 Mb, reaching a contig N50 of 11.97 Mb and containing 97.8% of complete BUSCOs. A total of 24,050 protein-coding genes were annotated, of which metalloproteinases, C-type lectins, and stonustoxins (*sntx*) were the most abundant putative toxin genes. Multitissue transcriptomic and venom proteomic data showed that *sntx* genes, especially those clustered within a 50-kb region on the chromosome 2, had higher transcription levels than other types of toxins as well as those *sntx* genes scatteringly distributed on other chromosomes. Further comparative genomic analysis predicted an expansion of *sntx*-like genes in the Percomorpha lineage including nonvenomous fishes, but *Scorpaenoidei* species experienced extra independent *sntx* duplication events, marking the clear-cut origin of authentic toxic stonustoxins. In summary, this high-quality genome assembly and related comparative analysis of toxin genes highlight valuable genetic differences for potential involvement in the evolution of venoms among *Scorpaeniformes* fishes.

## Introduction

It is estimated that there are over 2,900 species of venomous fish distributed throughout the world, and some of them such as stingrays, catfishes, and stonefishes have evolved venom glands in fins (Smith et al. 2016). Order Scorpaeniformes contains the most toxic species including stonefishes, scorpionfishes, and rockfishes. Among them, stonefishes, usually referring to the genus *Synanceia* with five extant species, are of highest venomous, and the representative reef stonefish (*Synanceia verrucosa*) is the most widely distributed species (Saggiomo et al. 2021). It has a complex venom composition with hemolytic (Ueda et al. 2006), coagulotoxic, and neurotoxic effects (Harris et al. 2021); thus the fish sting may result in a potentially lethal envenomation in human beings (Maillaud et al. 2020). Many experiments have also proved the toxicity of the fish venom in mice, rats, and dogs (Gail and Rageau 1956; Garnier et al. 1995; Khalil et al. 2018).

There have been many attempts to examine the complex venom composition of the reef stonefish as well as other toxic species in Scorpaeniformes (Saggiomo et al. 2021). This can trace back to the mid-90s, when a toxin was characterized in *Synanceia horrida* and named after the species name (stonustoxin [SNTX]) (Low et al. 1994; Poh et al. 1991). Later on, other toxins such as verrucotoxin (VTX, Garnier et al. 1995), neoVTX (Ueda et al. 2006), PaTx, PlTx, (Kiriake and Shiomi 2011), PvTx, IjTx, and HrTx (Kiriake et al. 2013) have also been identified from related species in the same order. Interestingly, although with different names and varied sequences, all these toxins may function as a heterodimer or tetramer composed of 2 homologous subunits (SNTX-α and SNTX-β) with similar molecular mass (70 to 80 kDa) as well as similar dimensional structures (Ellisdon et al. 2015; Saggiomo et al. 2021). Therefore, they were often called as stonefish toxin-like or SNTX-like toxins (Hatakeyama et al. 2021).

The 2 subunits of each SNTX toxin share sequence identify of ∼50%, but they both contain four domains, namely, membrane attack complex-perforin/cholesterol-dependent cytolysin (MACPF/CDC), focal adhesion-targeting (FAT), thioredoxin (THX), and PRY SPla and the Ryanodine Receptor (PRYSPRY). The α- and β-MACPF/CDC domains at the N-terminus interact to form a prepore-like complex, indicating that SNTXs may form pores in cells, and such a structure is essential for SNTX-induced pathologies and physiological effects (Ellisdon et al. 2015). SNTXs usually have hemolytic, myotoxic, vasorelaxant, and muscarinic activities in vitro and in vivo (Saggiomo et al. 2021), and inactivation studies show that these multiple activities can be inhibited by modification of anionic lipids, thiol groups, and some amino acid residues (such as lysine, arginine, and tryptophan) within the protein sequences (Saggiomo et al. 2021).

Besides the SNTX and its analogues, other toxins such as natterins (Hatakeyama et al. 2021), metalloproteases (Ziegman et al. 2019), and C-type lectins (Andrich et al. 2015) have also been identified in stonefishes and others in the same order, although most studies focused on only 1 or a few toxin(s) (Saggiomo et al. 2021). Recently, many omic approaches have been applied to comprehensively examine whole venom components of these species. For example, the venom composition of estuarine stonefish (*S. horrida*) has been investigated using an integrated transcriptomic and proteomic method (Ziegman et al. 2019). Xie et al. (2019) performed transcriptome analysis of venom glands from three scorpionfishes. However, none of these reports have investigated the venom component from a whole-genome view. Although some genomes of *Scorpaeniformes* species are available (He et al. 2019; Kolora et al. 2021), all of them are rockfishes (*Scorpaeniformes*: *Sebastidae*).

**Fig. 1.**
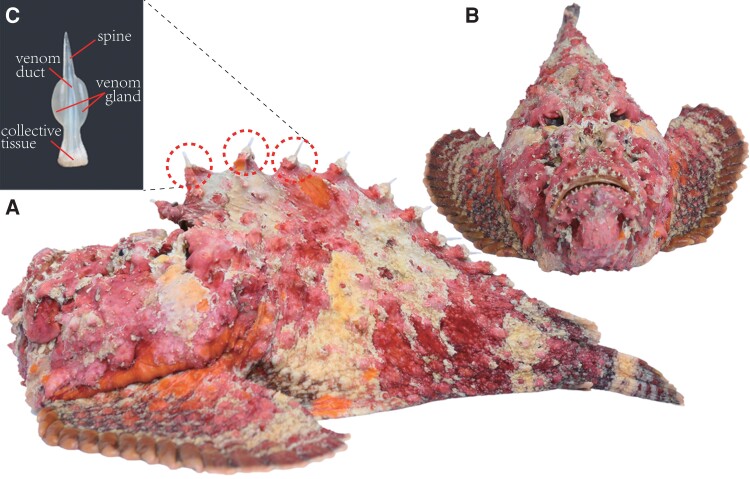
The sequenced reef stonefish and its venom gland. a) Lateral view of the fish. b) Head view of the fish. c) A representative venom gland dissected from the dorsal spine.

Our present study sequenced and assembled a chromosome (Chr)-level genome of the reef stonefish (Fig. 1), which is the first stonefish sequenced so far. This high-quality genome assembly makes inferring of the phylogeny of reef stonefish and its relatives available. Most importantly, combined with additional multitissue transcriptome and venom proteome data, this genomic resource prompts us to study the detailed repertoires, gene structures, and evolution of *sntx/sntx*-like toxins throughout the fish tree of life.

## Results

### Summary of the Genome Assembly and Annotations

After integrating 57-Gb Illumina short paired-end reads (Table S1) and 128-Gb Nanopore long reads (Table S2), we obtained a 689.52-Mb genome assembly of the reef stonefish containing 2,799 contigs with an N50 of 11.97 Mb (Table S3). This primary contig-level assembly was then improved with 131-Gb Hi-C reads (Table S4), resulting in 1,437 scaffolds with an N50 of 27.11 Mb (Table S5). The final Chr-level genome assembly reaches 689.74 Mb in length (Table 1), consistent with the 682.53-Mb genome size estimated by a k-mer analysis (Fig. S1).

**Table 1:** Summary of the genome assembly and gene annotation of the reef stonefish

Chr-Level Genome Assembly	Statistics
Total length (bp)	689,736,203
Contig N50 (bp)	12,009,665
Contig number (>100 bp)	2,230
Scaffold N50 (bp)	27,113,652
Scaffold number (>100 bp)	1,437
GC content	40.8%
**Gene Annotation**	**Statistics**
Total number of genes	24,050
Average mRNA length (bp)	10,929.40
Average CDS length (bp)	1,661.60
Average exon number per gene	9.08
Average exon length (bp)	182.95
Average intron length (bp)	1,146.72

**Fig. 2.**
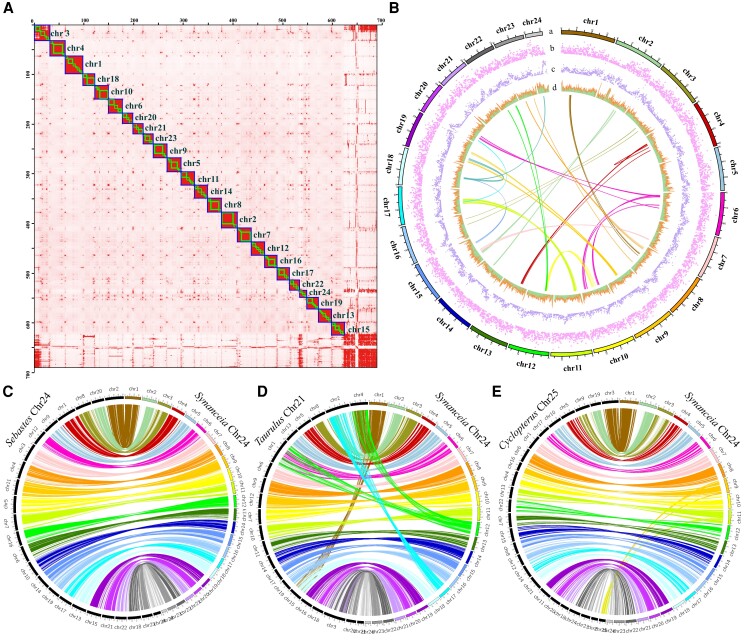
A Chr-level genome assembly of the reef stonefish with comparison to other related species. a) 24 distinct blocks were visualized in the Hi-C contact matrixes. b) The Chr-level genome assembly of the reef stonefish. From outside to inside: a) the 24 pseudo-chromosomes, b) gene density, c) repeats, and d) GC content. The links inside refer to internal syntenic blocks within the genome. c) Synteny between the Chr of the reef stonefish (2*n* = 48) and rockfish (2*n* = 48). d) Synteny between the Chr of the reef stonefish and sea scorpion (2*n* = 42). e) Synteny between the Chr of the reef stonefish and lumpfish (2*n* = 50).

A total of 628.82 Mb of the assembled sequences were clustered into 24 pseudo-Chr (Fig. 2A), ranging from 11.35 Mb to 35.82 Mb in length (Table S6). The GC (G for guanine and C for cytosine) content of the assembled reef stonefish genome was 40.8% (Table 1), similar to those of other reported *Scorpaeniformes* species such as the rockfish (Kolora et al. 2021; Liu et al. 2019). Genome completeness analysis identified 97.8% complete BUSCOs, consisting of 94.9% single-copy orthologs and 2.9% duplicates (Table S7). This high complete BUSCO score and the large contig N50 value indicate that the final genome assembly of the reef stonefish is of high quality, thereby qualifying for downstream analyses.

Subsequent repeats annotation identified 211 Mb of repetitive sequences, accounting for 30.59% of the reef stonefish genome (Table S8). The combined homology (using eight genomes in Table S9) and de novo annotation pipeline generates a total of 24,050 protein-coding genes, with an average mRNA length of 10,929 bp and nine exons for each gene (Table 1, Fig. S2). After blasting against various public databases (including SwissProt, TrEMBL, KEGG, and InterPro), 98.87% of the annotated genes have been predicted with at least 1 match in these databases (Table S10). Details of the Chr, gene density, GC content, repeat distribution, and internal synteny blocks are summarized in Fig. 2B to provide an overview of the reef stonefish genome.

### Chromosomal Evolution and Rearrangements

Most *Scorpaeniformes* species, including the reef stonefish are diploids with 24 pairs (2*n* = 48) of Chr; therefore, only few internal synteny links were observed in the assembled haploid genome of the reef stonefish (Fig. 2B). However, some related fishes have more or fewer Chr according to previous reports. For example, the long-spined sea scorpion (*Taurulus bubalis*) belonging to family Cottidae has 21 pairs of Chr (2*n* = 42; Potter 2021), while the common lumpfish (*Cyclopterus lumpus*) in Cyclopteridae has 25 pairs (2*n* = 50; Holborn et al. 2022). Comparison of the reef stonefish and honeycomb rockfish (from Sebastidae; 2*n* = 48) (Kolora et al. 2021) genomes shows a perfect one-to-one relationship between each pair of the Chr (Fig. 2C and S3A).

When compared to the sea scorpion genome, each of the Chr1, Chr2, and Chr3 of sea scorpion was determined to correspond to 2 Chr (Chr6 and 12, Chr2 and 17, and Chr21 and Chr22, respectively) of the reef stonefish (Figs. 2D and S3B). In contrast, 2 Chr (Chr16 and 25) of the lumpfish correspond to only 1 Chr (Chr9) of the stonefish (Figs. 2E and S3C). All these results suggest that these species from different families, with different Chr numbers, may have rearranged their Chr during the long-term evolution process.

### Construction of the Species Tree

**Fig. 3.**
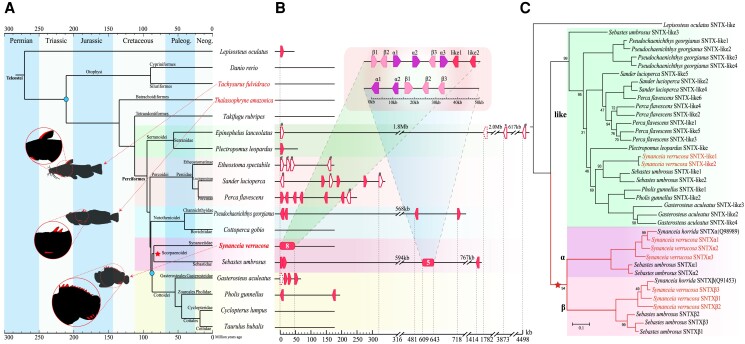
Phylogeny of 18 fish species and their stonustoxin family repertoires. a) Species tree with venomous species (highlighted in red) and their venomous spines. Each suborder branch in Perciformes is set to a different color background. b) Stonustoxin repertoires in various fishes. Each filled pentagon refers to a complete gene, while blank ones represent pseudogenes due to frameshifts (#) or pre-stop (*), and the dotted-lined ones denote partial genes. The 2 boxes refer to gene clusters. c) Phylogeny of the stonustoxins. Toxins from the reef stonefish were highlighted. The number at each node denotes the clade support, and those nodes without numbers have 100% bootstrap support.

Using the single-copy genes (Tables S11 to S12) identified from the whole genomes of reef stonefish and other 17 ray-finned fishes (Table S9) including spotted gar (as the outgroup), known venomous fishes, model species, and species in *Scorpaeniformes* and sister groups, we constructed a robust phylogenetic tree (Fig. 3A). Both the maximum likelihood (ML) and Bayesian inference (BI) methods established the same topology (Figs. S4 and S5), which is consistent with previous reports based on mitogenomes (Cui et al. 2019; Jia et al. 2020). Reef stonefish and honeycomb rockfish were grouped together, making up the Scorpaenoidei clade that is a sister group to the Cottoidei branch. The fossil-calibrated tree shows that the divergence time of reef stonefish and honeycomb rockfish was estimated to be around 84.5 million years ago (mya) in the Cretaceous period, which is similar to a previous estimate (Rabosky et al. 2018).

### Toxin Genes in the Reef Stonefish Genome

**Fig. 4.**
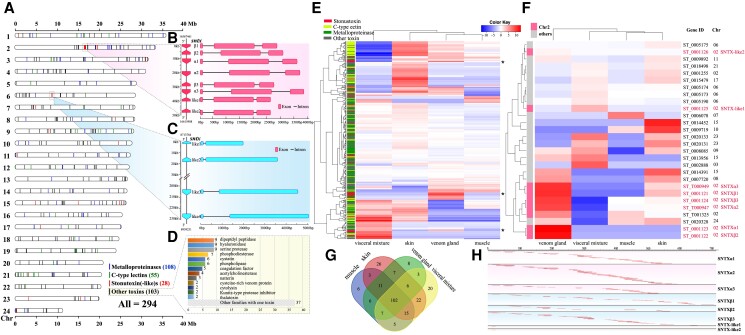
Chromosomal location and expression levels of the putative toxin genes of the reef stonefish. a) Localization of the putative toxin genes on the 24 Chr. Each dash represents a toxin gene. b) A cluster of 8 *sntx* genes located within a 50-kb region of the Chr2. Gene structure of each *sntx* is presented. c) Four *sntx*-like genes located on the Chr6. d) Classification of protein families for other 103 toxins. e) A heatmap of gene transcription with hierarchical clustering for all the 294 putative toxin genes. Three asterisks mark the highly expressed *sntx* genes in the venom. f) A heatmap of gene transcription with hierarchical clustering for the 28 *sntx* genes. Eight *sntx* genes clustered on the Chr2 were highlighted. The shared color key is provided, which shows low to high transcription levels from left to right. g) A Venn plot showing toxin genes transcribed in different tissues. h) Mapping of peptides from the venom proteome to the stonustoxins encoded from those sntx genes clustered on the Chr2. Long thick lines represent the stonustoxin proteins, and short thin lines stand for those mapped peptides in the proteome data.

A total of 294 putative toxin genes were annotated in the reef stonefish genome. However, they scattered throughout the 24 Chr (Fig. 4A). The most abundant toxins included metalloproteinases (108), C-type lectins (55), and SNTXs (28), which are similar to the reported venom content of estuarine stonefish (Ziegman et al. 2019). We also predicted other 103 toxins, including dipeptidyl peptidase (9), hyaluronidase (9), serine protease (9), phosphodiesterase (7), cystatin (6), phospholipase (6), coagulation factor (5), acetylcholinesterase (4), natterin (3), cysteine-rich venom protein (2), cytolysin (2), Kunitz-type protease inhibitor (2), thalatoxin (2), and other 37 families with only 1 toxin gene in each family (Fig. 4D).

Interestingly, 8 of the 28 *sntx* genes were clustered within a 50-kb region on Chr2 (Fig. 4B). They were similar in gene length, gene structure (with three exons), and encoded proteins with an amino acid length of around 700 (694 to 704), which are consistent with the length of known SNTX toxins (Yoshinaga-Kiriake et al. 2020).

However, the other annotated 20 *sntx*-like genes, scattered in different Chr, varied in both length and sequence. Most of them encoded only some of the 4 domains of a complete SNTX; thus these *sntx*-like genes were usually shorter than the normal one. However, there were few genes encoding proteins longer than a normal SNTX. For example, on the Chr6, there were 4 neighboring *sntx*-like genes, but all of them lost the third exon, with the second exon extended till the stop codon (Fig. 4C). In case they were false genes caused by incorrect annotation, we further checked the Iso-seq transcriptome and determined that the full-length transcripts of these genes existed.

### Expression of Toxin Genes in Various Tissues

Among the entire list of 294 putative toxin genes, 247 were detected by transcriptome sequencing (RNA-seq; see Table S13) with transcriptive evidence in at least one of the examined tissues (Fig. 4E), along with 126 novel toxin transcripts, which were found in the RNA-seq data, but did not exist in the gene annotation of the assembled genome. In detail, a total of 221 transcripts corresponding to 150 toxin genes were identified in the venom. Some putative toxin genes were also transcribed in nonvenom tissues, with 155, 192, and 180 toxin genes expressed in muscle, skin, and visceral mixture, respectively (Fig. 4G).

As expected, most of the detected *sntx* genes had high transcription levels in the venom gland (Fig. 4F), whereas other toxin gene families did not present such a pattern (Fig. S6). Interestingly, it is notable that a few *sntx* genes were also highly expressed in other tissues especially in the skin and visceral mixture, most likely because that spine-associated venom is hypothesized to have originated from the skin toxins (Harris and Jenner 2019) and that the liver and kidney in the visceral mixture are important organs for toxification and detoxification of xenobiotic compounds (Wolf and Wolfe 2005). It also suggests that these toxins may have other roles outside of envenomation. In addition, 6 of the 8 *sntx* genes clustered on the Chr2 generally had higher transcription levels in comparison with those on other Chr (in a scattered way), suggesting that these toxins encoded by *sntx* genes clustered on the Chr2 might be the core components of the reef stonefish venom.

This hypothesis was further supported by a proteomic analysis of the venom gland. A total of 3,587 peptides, corresponding to 1,076 proteins, were identified using the deduced protein sequences from the assembled genome as the reference. Among these detected peptides, 194 sequences were toxin fragments, including 127, 17, 11, and 39 peptides from SNTXs, lectins, metalloproteinases, and other toxins, respectively. As expected, the majority (22 peptides) of the top 30 peptides with the highest intensity were digested from the SNTX proteins (Table S14). In addition, up to 191 peptides (including multiple mapped peptides) were mapped to the eight SNTXs encoded from the *sntx* genes located on the Chr2. Similar to the transcription pattern in the venom transcriptome (Fig. 4F), almost all of these peptides biasedly hit the 6 genes with high expression levels (Fig. 4H), with only 1 peptide mapped to each of the 2 SNTX-likes. Such finding not only proves that SNTXs coming from the Chr2 probably serve as the major bioactive toxins in the venom but also illuminates that some of them might be active while others are not for the toxic effects.

### Stonustoxin Evolution Throughout the Fish Tree of Life

To investigate *sntx* evolution throughout the fish tree of life, we also predicted the *sntx* repertoires from other 17 representative species in addition to the reef stonefish, using the reviewed SNTX subunits as references to search against the whole genomes of those corresponding fishes. The retrieved protein sequences that were too long (>120%) or too short (<80%) were eliminated. The results show that, with a *sntx* cluster of 8 genes on the Chr2, the reef stonefish encodes the largest repertoire of SNTXs as expected, followed by the honeycomb rockfish (encoding 8 SNTXs in total, 5 of which were clustered together as well). Such clusters could only be identified in the 2 *Scorpaenoidei* species, while the *sntx* genes were tandemly located in other fish genomes (Fig. 3B). It seems that there might be an expansion of *sntx* genes in the *Scorpaenoidei* fishes, which has been discussed in previous reports (such as Ellisdon et al. 2015).

The phylogeny of SNTX proteins split them into 2 major clades, using the SNTX of spotted gar as the outgroup. A small clade (the αβ clade) includes the 2 reported SNTX subunits, the 6 highly expressed stonefish SNTXs from the Chr2, and all 5 clustered SNTXs in the rockfish. It was further divided into 2 groups, 1 represents the α subunit, and the other denotes the β subunit. As a sister to the small clade, the big clade consists of all the SNTXs from other non-*Scorpaenoidei* species, the 2 lowly expressed stonefish SNTXs from the Chr2, as well as the 3three unclustered SNTXs in the rockfish (Fig. 3C). This interesting phylogeny implies that although SNTXs exist in both venomous and nonvenomous fishes (Ellisdon et al. 2015), the SNTX-α and SNTX-β subunits could only be well defined in *Scorpaenoidei* fishes (Ellisdon et al. 2015; Hatakeyama et al. 2021; Yoshinaga-Kiriake et al. 2020), suggesting that the neofunction of duplicated SNTXs as toxins might have occurred after the divergence of Scorpaenoidei and Cottoidei.

## Discussion

### Valuable Genome Assembly of the Venomous Reef Stonefish

Rapid development of sequencing technologies and bioinformatics facilitates whole-genome assembling and downstream analysis, and there are more than 900 fish records deposited in the NCBI Genome database so far. However, venomous fishes have been given little attention, with only a few species such as yellow catfish (Zhang et al. 2018) and prehistoric monster fish were sequenced till now. Scorpaenoidei contains the most venomous fishes, but only the mildly toxic rockfish genomes are available (He et al. 2019; Kolora et al. 2021), while their venoms are scarcely studied (Francisco et al. 2021). Our present study constructed a Chr-level genome assembly of the reef stonefish, representative of the venomous stonefishes whose venom components have been widely studied (Ellisdon et al. 2015). This high-quality genome assembly not only makes the extensive genetic research of stonefish toxin genes available but also provides a fundamental genetic resource for in-depth biomedical study of this venomous species.

### Unique Venom Components of the Reef Stonefish

Fish venom systems are supposed to be evolved convergently 19 times (Harris and Jenner 2019), while bioactive compounds in venom glands are different. For instance, the yellow catfish venom contains veficolin, ink toxin, adamalysin, Za2G, and CRISP toxin (Xie et al. 2016; Zhang et al. 2018), but the major components of the toadfish venom are natterin (Lima et al. 2021) and tetrodotoxin, a well-known toxin found in pufferfish (Katikou et al. 2022). None of these venomous fishes have been reported to contain any SNTX toxin, which is consistent with our current analysis of the SNTX repertoires throughout fish tree of life (Fig. 3B).

In addition to SNTXs, there are hundreds of other toxins such as metalloproteinases and C-type lectins in the reef stonefish genome (Fig. 4), similar to those reports in estuarine stonefish using both transcriptomic and proteomic methods (Ziegman et al. 2019). However, *sntx* genes, especially those clustered on the Chr2, generally had higher expression levels in the venom gland, compared to other types of toxin genes (Fig. 4E and S6), as well as the rest of the *sntx* genes scattered on other Chr (Fig. 4F), both at the transcriptional and translational levels (Table S14). Taken together, this special toxin gene distribution and expression pattern suggest that SNTXs are potentially the pivotal bioactive compounds in the venom of reef stonefish and even of all the venomous *Scorpaenoidei* species when combined with previous reports on the venoms of other stonefishes (Ziegman et al. 2019), scorpionfish (Xie et al. 2019), waspfish (Kiriake et al. 2013), and rabbitfish (Kiriake et al. 2017).

### Multiple Copies of *sntx* Genes in the Reef Stonefish Genome

Unexpectedly, there were more than 1 pair of SNTX subunits (α and β) in the reef stonefish genome. The number totaled 28 (Fig. 4A), of which 8 genes were clustered within a 50 kb-region on the Chr2 (Fig. 4B). In contrast to the rest of these SNTXs with variable lengths, all the 8 SNTXs were composed of about 700 amino acid residues, resembling those reported SNTXs (Ziegman et al. 2019).

Both phylogeny (Fig. 3C, Table S12) and protein sequences (Fig. S7, Table S15) classified the 8 SNTXs into 3 SNTX-α subunits, 3 SNTX-β subunits, and 2 SNTX-like proteins. The SNTX-α and -β copies had higher expressions in the venom than the SNTX-like proteins (Fig. 4F to H). Although SNTX-like proteins had similar gene structures and the same 4 domains as normal SNTXs (Ziegman et al. 2019), some conserved sites mutated into other amino acids, such as the SNTX-α/β-interacting residues in the focal adhesion-targeting domain (Fig. S7). In summary, our results imply that the 3 pairs of SNTX-α/β might function as bioactive toxins in the venom gland, while the SNTX-like proteins probably do not; this potential is similar to those SNTXs identified in other nonvenomous species (Fig. 3C; Ellisdon et al. 2015).

The 3D structure of SNTX from stonefish *S. horrida* was described before (Ellisdon et al. 2015) using X-ray crystallography (as shown in Fig. S8A). The 8 SNTXs found in our present study have similar 3D structural characteristics with SNTX-α and SNTX-β from the stonefish *S. horrida* using homologous modeling (Fig. S8B). In particular, the sequence identity of 2 SNTX-like proteins is only about 48.16% and 46.80%, respectively (Table S15), but they still present similar 3D structures, suggesting that SNTX-like and SNTX-αβ proteins have the same origin.

### Interesting SNTX Evolution Throughout the Fish Tree of Life

It is estimated that SNTXs were generated by gene duplication multiple times throughout the evolutionary history of Percomorpha fish (Ellisdon et al. 2015). Similarly, we detected an expansion of *sntx* genes in the Percomorpha lineage, while with gene loss evidence in several other branches (Fig. 3B). In addition, extra gene duplications occurred in the Scorpaenoidei fish, and these events may have led to the generation of authentic toxic SNTXs by neo-functionalization (Magadum et al. 2013). Our phylogenetic analysis also implies that these extra gene duplication events happened after the divergence of the 2 genera of *Synanceia* and *Sebastes*, since the subunits from the same genus grouped together to form sister groups within each clade of both subunits (Fig. 3C). However, it is unclear whether these SNTX duplicates including those less conserved SNTX-like proteins form right dimers for function or not, and the detailed process of the duplication events is still unknown yet. More experiments and additional genomic studies are required to address these unresolved questions.

## Materials and Methods

### Sample Collection, Preparation, and Species Identification

A wild reef stonefish was collected from Xisha Islands in the South China Sea with official permission. The fish was temporarily cultured in a seawater tank and then anesthetized before sample preparation. Genomic DNA was extracted from the muscle for whole-genome sequencing, and total RNA was extracted from muscle, venom gland, skin, and liver for transcriptome sequencing. DNA and RNA quantity, purity, and integrity were assessed by qubit fluorometer, NanoDrop spectrophotometer and gel electrophoresis, respectively.

Species identification was carried out by both morphologic determination and complete mitochondrial genome assembled from raw genomic reads using SPAdes v3.13.0 with parameters -k 71,73,75,77 (Prjibelski et al. 2020). Animal experiments in this study were reviewed and approved by the Institutional Review Board on Bioethics and Biosafety of BGI (Shenzhen, China).

### Genome and Transcriptome Sequencing

We integrated Illumina short reads, Nanopore long reads, and Hi-C data to establish a high-quality genome assembly of the reef stonefish. For the short-read sequencing, a library with an insert size of 350 bp was constructed and sequenced on an Illumina Hiseq X-Ten platform (Illumina Inc., San Diego, CA, USA) to generate 2 × 150-bp paired-end raw reads, which were further filtered by SOAPnuke v2.1.0 (−z -p -M 2 -trim [5,0,5,0]) (Chen et al. 2018). For the long-read sequencing, a library with a 20-kb insert size was prepared and sequenced by MinION of Oxford Nanopore Technologies (ONT; Nanopore Technologies, Oxford, UK). For the Hi-C sequencing, a library with a 400-bp insert size was constructed using cross-linked chromatin fragments (Ghurye et al. 2019) and sequenced on an Illumina Hiseq X-Ten platform.

For transcriptome sequencing, RNA-seq libraries were constructed separately for each of the 4 different samples including venom gland, muscle, skin, and visceral mixture (refers to the mixture of internal tissues, including the liver, spleen, kidney, and heart) and sequenced on an Illumina Hiseq X-Ten platform. A full-length transcriptome sequencing of the venom gland was parallelly conducted on an Iso-seq PacBio platform (Pacific Biosciences, Menlo Park, CA, USA).

### Genome Size Estimation and De Novo Genome Assembly

To estimate the genome size of the reef stonefish, the k-mer frequencies were calculated for those genomic short reads with GCE v1.0.2 (Liu et al. 2013). To obtain a high-quality Chr-level genome assembly, we first assembled clean short reads into contigs using Platanus v1.2.4 (-k 29 -d 0.3 -t 16 -m 300) (Kajitani et al. 2019) and then aligned them against the ONT long reads with DBG2OLC (Ye et al. 2016) to generate a consensus assembly, which was subjected to 3 iterations of polishing by NextPolish v1.4.0 (Hu et al. 2020). Subsequently, HiC-Pro v2.2 (Servant et al. 2015) was employed for quality control of the Hi-C raw data, and those valid reads were mapped to the polished assembly for scaffolding contigs by using Juicer v1.5 (Durand, Shamim, et al. 2016) plus the 3D-DNA v180922 pipeline (Dudchenko et al. 2017). Finally, the pseudo-chromosomal assembly was visualized and polished manually with Juicebox (Durand, Robinson, et al. 2016) according to the contact maps and boundaries. Completeness of the final genome assembly was assessed by BUSCO v5.2.2 with genome mode and the database actinopterygii_odb9 (Simão et al. 2015).

### Genome Annotation and Gene Prediction

Repeats in the genome were annotated by both de novo and homology methods. For the de novo prediction, RepeatModeler v1.0.11 (Flynn et al. 2020) and LTR-FINDER v4.09.1 (Xu and Wang 2007) were applied to construct 2 independent repeat libraries, which were subsequently combined and aligned to the final genome assembly with RepeatMasker v1.331 (Tarailo-Graovac and Chen 2009). For the homology prediction, RepeatMasker and RepeatProteinMask were employed with the known Repbase library (Jurka et al. 2005). In addition, Tandem Repeats Finder (TRF) v4.07b (Benson 1999) was employed to detect tandem repeats. Finally, overlapping repeats from different methods were integrated to generate the final nonredundant repeat repertoire.

To annotate gene structures, we applied the following three approaches. First, for homology search, protein sequences of 8 representative bony fishes were downloaded from the NCBI database and then used as references to get the reef stonefish's gene structures using TBLASTn (Gertz et al. 2006) followed by GeneWise (Birney et al. 2004). Second, for transcriptome-based prediction, the RNA-seq short reads were mapped onto the assembled genome by using HISAT2 v2.0.4 (Kim et al. 2019), and then the alignments were used to predict gene structures with Cufflinks v2.2.1 (Trapnell et al. 2010). Third, for ab initio prediction, a transcriptome assembly was de novo assembled by Trinity v2.13.2 (Grabherr et al. 2011), which was used as the training set for gene prediction by Augustus v3.3.1 (Stanke et al. 2006). Finally, MAKER 3.01.01 (Cantarel et al. 2008) was employed to integrate all data generated from the abovementioned 3 methods so as to obtain the final consistent gene set. Gene function and protein domain/motif information were then obtained by blasting the deduced protein sequences against several public databases including NCBI nonredundant protein (nr), SwissProt (Boeckmann et al. 2003), Kyoto Encyclopedia of Genes and Genomes (KEGG) (Kanehisa 2000), gene ontology (GO) (Harris et al., 2004), and InterPro (Jones et al. 2014).

### Intraspecific and Interspecific Chromosomal Comparisons

We performed both intraspecific and interspecific chromosomal comparisons between the reef stonefish and other related fish species. For the intraspecific comparison, the protein set of reef stonefish was used as both the subject and the query for BLASTp search, and the alignments were fed into MCscan (Wang et al. 2012) with parameters “-a -e 1e-5 -u 1 -s 8” to construct genome internal syntenic blocks. For the interspecific comparison, the assembled genomes of reef stonefish and other bony fishes were aligned by using Lastz v1.04.15 with parameters “T = 2 C = 2 H = 2,000 Y = 3,400 L = 6,000 K = 2,200.” The Circos plots were drawn by Circos v0.69.3 (Krzywinski et al. 2009), and the dot plots were prepared by the LAST v 973 package (Kiełbasa et al. 2011).

### Annotation of *sntx*/*sntx*-Like Genes and Other Toxin Genes

Toxin annotation was based on the above-described functional gene annotation procedure. We retrieved those genes annotated as “toxin” or “venom” but without “receptor.” Subsequently, the most predominant venom proteins (including metalloproteinases, C-type lectins, and stonustoxins) reported in stonefish before (Ziegman et al. 2019) were also selected. Meanwhile, genes enriched into the GO term “toxin activity” (GO:0090729) were picked out. Finally, all results were merged without duplicates to generate the final nonredundant toxin gene set. In this study, putative toxin genes refer to those identified in the assembled genome, no matter they existed in the transcriptome or proteome of the venom or not, since some toxin transcripts or peptides will certainly be lost during sample and data processing or due to low sensitivity of detection machines.

Gene structures of the SNTX-like toxins were further verified by Exonerate v2.2.0 (Slater and Birney 2005). A synteny analysis of these *sntx*-like genes and their neighboring genes (both upstream and downstream) was first performed by blasting against each target genome independently with the stonefish proteins as the references. The best hit for each blast search was selected, and then the gene arrangement and location were plotted by using the SVG module in Perl.

### Evolutionary Analysis

To construct the phylogenetic tree, we firstly applied OrthoFinder v2.2.7 (Emms and Kelly 2019) to identify single-copy gene families from the assembled genomes of reef stonefish and 17 other fishes (including spotted gar as the outgroup), which were downloaded from the NCBI Genome database. Subsequently, coding sequence (CDS) of each gene family was aligned with MUSCLE v5.1 (Edgar 2004) and concatenated, and then the 1st codon site was selected and the final alignment was trimmed with Gblocks (Castresana 2000). A ML tree was constructed using PhyML v3.0 (Guindon et al. 2010) with the GTR substitution model selected by ModelFinder implemented in IQ-TREE v1.6.12 (Nguyen et al. 2015). Branch supports were evaluated by the approximate likelihood ratio test (aLRT) (Anisimova and Gascuel 2006). We also built a BI tree using MrBayes v3.2.2 (Ronquist et al. 2012) with the GTR + I + G model selected by MrModeltest v2.4 implemented in PAUP v4 (www.paup.phylosolutions.com), by running 100,000 generations with sampling every 100 generations. The initial 20% runs were discarded as burn-in. Finally, the 2 species trees were compared, and divergence times were estimated by the Bayesian method implemented by MCMCtree in PAML v4.9 (Yang 2007), using a relaxed-clock model with the following 2 fossil times (may), including (i) Otophysi–Batrachoidiformes: 180.0 to 264.0, and (ii) Scorpaenoidei-Cottoidei: 64.7 to 110.2, from TimeTree (Kumar et al. 2017) as constraints. We ran a total of 100,000 samples for the MCMC analysis, of which the first 20% were discarded as burn-in.

Evolution analysis of the *sntx*-like toxins was performed using their protein sequences. Those SNTX-like toxin protein sequences were aligned using MAFFT v7.490 (Rozewicki et al. 2019). ModelFinder (Kalyaanamoorthy et al. 2017) was applied to determine the best-fit substitution model (JTT + F + R4), and then a ML tree was constructed using IQ-TREE v1.6.12 (Nguyen et al. 2015) with node support assessments by running 1,000 bootstrap replications.

### Gene Transcriptional Quantification and Differential Expression Analysis

To calculate gene transcription levels, bowtie2 v2.4.0 (Langmead and Salzberg 2012) was applied to map the quality-controlled RNA-seq clean reads to the assembled reef stonefish genome. Then the aligned reads were counted, and the gene transcription was quantified by RSEM v1.3.3 (Li and Dewey 2011). Subsequently, differently expressed genes (DEGs) were identified by DESeq2 v1.26.0 (Love et al. 2014) with an adjusted *P* < 0.05 and a log-fold change ≥ 2. Finally, the toxin DEGs across all samples were visualized by the pheatmap package v1.0.12 in R with normalized values via *Z* score.

### Proteome Profiling of the Venom

Crude venom (about 300 µL) was collected from all the dorsal spines of the same sequenced fish by squeezing the spines against the cling wrap sealed over a 1.5 mL tube. For proteome profiling, the sample was first prepared according to the filter aided sample preparation (FASP) procedure (Wiśniewski 2019). In brief, we used 10 µL of the crude venom and added dithiothreitol to dilute the solution to a final concentration of 100 mM. It was then centrifugated, and the pellet was washed twice with 8 M urea in 0.1 M Tris/HCl pH 8.5 (UA buffer), incubated with 100 mM iodoacetamide (IAA buffer) at room temperature for 30 min in dark, and then washed twice by UA buffer again. Afterward, the pelleted was resolved in 100 µL of 25 mM ammonium bicarbonate for centrifugation, followed by trypsinolysis at 37 °C for 18 h with 40 µL of trypsin buffer (4 µg trypsin in 40 µL of 100 mM ammonium bicarbonate). Finally, 10 µL of acetic acid was added to stop the reaction, and the solution was further submitted to solid-phase extraction on C18 cartridges to obtain the final desalted peptide fragments.

LC-MS/MS detection was performed on a prominence nano-HPLC system (Shimadzu, Tokyo, Japan). Peptides were initially trapped on a column (100 µm × 2 cm, 5 µm-C18), and then isolated on a column (75 µm × 100 mm, 3 µm-C18) at a flow rate of 300 nL/min. Next, spectral data were analyzed by using Q-Exactive (Thermo Fisher Scientific, Waltham, MA, USA), with settings of analyzing time (120 min), parent ion scan range (300 to 1800 m/z), ms1 resolving power (70,000 at m/z 200), AGC target (3e6), and ms1 maximum IT (50 ms). Identified ions were further fragmented and analyzed with settings of ms2 resolving power (17,500 at m/z 200), isolation window (2 m/z), ms2 maximum IT (60 ms), ms2 activation type (HCD), normalized collision energy (27 eV), dynamic exclusion (60.0 s), and underfill ratio (0.1%). Raw files were fed into MaxQuant (Tyanova et al. 2016) for searching the protein library predicted from the assembled genome and transcriptomes of reef stonefish with parameters (enzyme: trypsin, missed cleavage sites: 2, fixed modification: C, variable modification: M and protein N-term acetyl), and those peptides with false discovery rates (FDR) ≤ 0.01 were considered reliable. Peptide intensities were calculated as the sums of extracted ion current (XIC) from all isotopic clusters associated with the identified peptide; in other words, they show the sums of all identified individual (both unique and razor) peptide intensities.

### 3D-homology Model of SNTX-Like Toxins

The 3D structure models of SNTX-like toxins were developed from primary amino acid sequences via homology modeling on a SWISS-MODEL server (Waterhouse et al. 2018). Models of the SNTX-like toxins were built using Stonustoxin (PDB:4WVM) including subunit alpha and beta Stonustoxin structures as templates in the SWISS-MODEL server, and related QMEAN scores were applied to evaluate the model quality.

## Supplementary Materials

Supplementary material is available at *Molecular Biology and Evolution* online.

## Supplementary Material

msad215_Supplementary_Data

## Data Availability

All data including the genome assembly and the raw reads generated by the present study are available at NCBI with accession number PRJNA930159.

## References

[msad215-B1] Andrich F , Richardson M, Naumann GB, Cordeiro MN, Santo AV, Santos DM, Oliveira JS, de Lima ME, Figueiredo SG. Identification of C-type isolectins in the venom of the scorpionfish *Scorpaena plumieri*. Toxicon. 2015:95:67–71. 10.1016/j.toxicon.2015.01.004. 25576236

[msad215-B2] Anisimova M , Gascuel O. Approximate likelihood-ratio test for branches: a fast, accurate, and powerful alternative. Syst Biol. 2006:55(4):539–552. 10.1080/10635150600755453. 16785212

[msad215-B3] Benson G . Tandem Repeats Finder: a program to analyze DNA sequences. Nucleic Acids Res. 1999:27(2):573–580. 10.1093/nar/27.2.573. 9862982 PMC148217

[msad215-B4] Birney E , Clamp M, Durbin R. Genewise and genomewise. Genome Res. 2004:14(5):988–995. 10.1101/gr.1865504. 15123596 PMC479130

[msad215-B5] Boeckmann B , Bairoch A, Apweiler R, Blatter MC, Estreicher A, Gasteiger E, Marti MJ, Michoud K, O’Donovan C, Phan I, et al The SWISS-PROT protein knowledgebase and its supplement TrEMBL in 2003. Nucleic Acids Res. 2003:31(1):365–370. 10.1093/nar/gkg095. 12520024 PMC165542

[msad215-B6] Cantarel BL , Korf I, Robb SMC, Parra G, Ross E, Moore B, Holt C, Sánchez Alvarado A, Yandell M. MAKER: an easy-to-use annotation pipeline designed for emerging model organism genomes. Genome Res. 2008:18(1):188–196. 10.1101/gr.6743907. 18025269 PMC2134774

[msad215-B7] Castresana J . Selection of conserved blocks from multiple alignments for their use in phylogenetic analysis. Mol Biol Evol. 2000:17(4):540–552. 10.1093/oxfordjournals.molbev.a026334. 10742046

[msad215-B8] Chen Y , Chen Y, Shi C, Huang Z, Zhang Y, Li S, Li Y, Ye J, Yu C, Li Z, et al SOAPnuke: a MapReduce acceleration-supported software for integrated quality control and preprocessing of high-throughput sequencing data. GigaScience. 2018:7(1):1–6. 10.1093/gigascience/gix120. PMC5788068 29220494

[msad215-B9] Cui L , Cao R, Dong Y, Gao X, Cen J, Lu S. The first complete mitochondrial genome of the flathead *Cociella crocodilus* (Scorpaeniformes: Platycephalidae) and the phylogenetic relationships within Scorpaeniformes based on whole mitogenomes. Genes (Basel). 2019:10(7):533. 10.3390/genes10070533. 31311107 PMC6678826

[msad215-B10] Dudchenko O , Batra SS, Omer AD, Nyquist SK, Hoeger M, Durand NC, Shamim MS, Machol I, Lander ES, Aiden AP, et al De novo assembly of the *Aedes aegypti* genome using Hi-C yields chromosome-length scaffolds. Science. 2017:356(6333):92–95. 10.1126/science.aal3327. 28336562 PMC5635820

[msad215-B11] Durand NC , Robinson JT, Shamim MS, Machol I, Mesirov JP, Lander ES, Aiden EL. Juicebox provides a visualization system for Hi-C contact maps with unlimited zoom. Cell Syst. 2016:3(1):99–101. 10.1016/j.cels.2015.07.012. 27467250 PMC5596920

[msad215-B12] Durand NC , Shamim MS, Machol I, Rao SSP, Huntley MH, Lander ES, Aiden EL. Juicer provides a one-click system for analyzing loop-resolution Hi-C experiments. Cell Syst. 2016:3(1):95–98. 10.1016/j.cels.2016.07.002. 27467249 PMC5846465

[msad215-B13] Edgar RC . MUSCLE: multiple sequence alignment with high accuracy and high throughput. Nucleic Acids Res. 2004:32(5):1792–1797. 10.1093/nar/gkh340. 15034147 PMC390337

[msad215-B14] Ellisdon AM , Reboul CF, Panjikar S, Huynh K, Oellig CA, Winter KL, Dunstone MA, Hodgson WC, Seymour J, Dearden P, et al Stonefish toxin defines an ancient branch of the perforin-like superfamily. Proc Natl Acad Sci USA. 2015:112(50):15360–15365. 10.1073/pnas.1507622112. 26627714 PMC4687532

[msad215-B15] Emms DM , Kelly S. OrthoFinder: phylogenetic orthology inference for comparative genomics. Genome Biol. 2019:20(1):238. 10.1186/s13059-019-1832-y. 31727128 PMC6857279

[msad215-B16] Flynn JM , Hubley R, Goubert C, Rosen J, Clark AG, Feschotte C, Smit AF. RepeatModeler2 for automated genomic discovery of transposable element families. Proc Natl Acad Sci USA. 2020:117(17):9451–9457. 10.1073/pnas.1921046117. 32300014 PMC7196820

[msad215-B17] Francisco SM , Castilho R, Lima CS, Almada F, Rodrigues F, Šanda R, Vukić J, Pappalardo AM, Ferrito V, Robalo JI. Genetic hypervariability of a Northeastern Atlantic venomous rockfish. PeerJ. 2021:9:e11730. 10.7717/peerj.11730. 34306828 PMC8280884

[msad215-B18] Gail R , Rageau J. Premières observations sur un poisson marin venimeux de la Nouvelle- Calédonie: La synancée (*Synanceia verrucosa* Bloch) [first findings on a venomous marine fish from New Caledonia, the stonefish Synanceia verrucos]. Bull Soc Pathol Exot. 1956:49(5):846–854. 13413617

[msad215-B19] Garnier P , Goudey-Perrière F, Breton P, Dewulf C, Petek F, Perrière C. Enzymatic properties of the stonefish (*Synanceia verrucosa* Bloch and Schneider, 1801) venom and purification of a lethal, hypotensive and cytolytic factor. Toxicon. 1995:33(2):143–155. 10.1016/0041-0101(94)00151-W. 7597718

[msad215-B20] Gertz EM , Yu YK, Agarwala R, Schäffer AA, Altschul SF. Composition-based statistics and translated nucleotide searches: improving the TBLASTN module of BLAST. BMC Biol. 2006:4(1):41. 10.1186/1741-7007-4-41. 17156431 PMC1779365

[msad215-B21] Ghurye J , Rhie A, Walenz BP, Schmitt A, Selvaraj S, Pop M, Phillippy AM, Koren S. Integrating Hi-C links with assembly graphs for chromosome-scale assembly. PLoS Comput Biol. 2019:15(8):e1007273. 10.1371/journal.pcbi.1007273. 31433799 PMC6719893

[msad215-B22] Grabherr MG , Haas BJ, Yassour M, Levin JZ, Thompson DA, Amit I, Adiconis X, Fan L, Raychowdhury R, Zeng Q, et al Full-length transcriptome assembly from RNA-seq data without a reference genome. Nat Biotechnol. 2011:29(7):644–652. 10.1038/nbt.1883. 21572440 PMC3571712

[msad215-B23] Guindon S , Dufayard JF, Lefort V, Anisimova M, Hordijk W, Gascuel O. New algorithms and methods to estimate maximum-likelihood phylogenies: assessing the performance of PhyML 3.0. Syst Biol. 2010:59(3):307–321. 10.1093/sysbio/syq010. 20525638

[msad215-B24] Harris MA , Clark J, Ireland A, Lomax J, Ashburner M, Foulger R, Eilbeck K, Lewis S, Marshall B, Mungall C, et al The gene ontology (GO) database and informatics resource. Nucleic Acids Res. 2004:32(Database issue):258–261. 10.1093/nar/gkh036. PMC308770 14681407

[msad215-B25] Harris RJ , Jenner RA. Evolutionary ecology of fish venom: adaptations and consequences of evolving a venom system. Toxins (Basel). 2019:11(2):60. 10.3390/toxins11020060. 30678265 PMC6409815

[msad215-B26] Harris RJ , Youngman NJ, Chan W, Bosmans F, Cheney KL, Fry BG. Getting stoned: characterisation of the coagulotoxic and neurotoxic effects of reef stonefish (*Synanceia verrucosa*) venom. Toxicol Lett. 2021:346:16–22. 10.1016/j.toxlet.2021.04.007. 33878385

[msad215-B27] Hatakeyama T , Kishigawa A, Unno H. Molecular cloning and characterization of the two putative toxins expressed in the venom of the devil stinger *Inimicus japonicus*. Toxicon. 2021:201:9–20. 10.1016/j.toxicon.2021.08.006. 34391787

[msad215-B28] He Y , Chang Y, Bao L, Yu M, Li R, Niu J, Kong X, Peng M, Sun M, Wang M, et al A chromosome-level genome of black rockfish, *Sebastes schlegelii*, provides insights into the evolution of live birth. Mol Ecol Resour. 2019:19(5):1309–1321. 10.1111/1755-0998.13034. 31077549

[msad215-B29] Holborn MK , Einfeldt AL, Kess T, Duffy SJ, Messmer AM, Langille BL, Brachmann MK, Gauthier J, Bentzen P, Knutsen TM, et al Reference genome of lumpfish *Cyclopterus lumpus* Linnaeus provides evidence of male heterogametic sex determination through the AMH pathway. Mol Ecol Resour. 2022:22(4):1427–1439. 10.1111/1755-0998.13565. 34859595

[msad215-B30] Hu J , Fan J, Sun Z, Liu S. NextPolish: a fast and efficient genome polishing tool for long-read assembly. Bioinform. 2020:36(7):2253–2255. 10.1093/bioinformatics/btz891. 31778144

[msad215-B31] Jia C , Zhang X, Xu S, Yang T, Yanagimoto T, Gao T. Comparative analysis of the complete mitochondrial genomes of three rockfishes (Scorpaeniformes. Sebastiscus) and insights into the phylogenetic relationships of Sebastidae. Biosci Rep. 2020:40(12):BSR20203379. 10.1042/BSR20203379. 33245090 PMC7736627

[msad215-B32] Jones P , Binns D, Chang HY, Fraser M, Li W, McAnulla C, Maslen J, Mitchell A, Nuka G, Pesseat S, et al Interproscan 5: genome-scale protein function classification. Bioinformatics. 2014:30(9):1236–1240. 10.1093/bioinformatics/btu031. 24451626 PMC3998142

[msad215-B33] Jurka J , Kapitonov VV, Pavlicek A, Klonowski P, Kohany O, Walichiewicz J. Repbase update, a database of eukaryotic repetitive elements. Cytogenet Genome Res. 2005:110(1-4):462–467. 10.1159/000084979. 16093699

[msad215-B34] Kajitani R , Yoshimura D, Okuno M, Minakuchi Y, Kagoshima H, Fujiyama A, Kubokawa K, Kohara Y, Toyoda A, Itoh T. Platanus-allee is a de novo haplotype assembler enabling a comprehensive access to divergent heterozygous regions. Nat Commun. 2019:10(1):1702. 10.1038/s41467-019-09575-2. 30979905 PMC6461651

[msad215-B35] Kalyaanamoorthy S , Minh BQ, Wong TKF, von Haeseler A, Jermiin LS. Modelfinder: fast model selection for accurate phylogenetic estimates. Nat Methods. 2017:14(6):587–589. 10.1038/nmeth.4285. 28481363 PMC5453245

[msad215-B36] Kanehisa M . KEGG: Kyoto Encyclopedia of Genes and Genomes. Nucleic Acids Res. 2000:28(1):27–30. 10.1093/nar/28.1.27. 10592173 PMC102409

[msad215-B37] Katikou P , Gokbulut C, Kosker AR, Campàs M, Ozogul F. An updated review of tetrodotoxin and its peculiarities. Mar Drugs. 2022:20(1):47. 10.3390/md20010047. 35049902 PMC8780202

[msad215-B38] Khalil AM , Wahsha MA, Abu Khadra KM, Khalaf MA, Al-Najjar TH. Biochemical and histopathological effects of the stonefish (*Synanceia verrucosa*) venom in rats. Toxicon. 2018:142:45–51. 10.1016/j.toxicon.2017.12.052. 29294314

[msad215-B39] Kiełbasa SM , Wan R, Sato K, Horton P, Frith MC. Adaptive seeds tame genomic sequence comparison. Genome Res. 2011:21(3):487–493. 10.1101/gr.113985.110. 21209072 PMC3044862

[msad215-B40] Kim D , Paggi JM, Park C, Bennett C, Salzberg SL. Graph-based genome alignment and genotyping with HISAT2 and HISAT-genotype. Nat Biotechnol. 2019:37(8):907–915. 10.1038/s41587-019-0201-4. 31375807 PMC7605509

[msad215-B41] Kiriake A , Ishizaki S, Nagashima Y, Shiomi K. Occurrence of a stonefish toxin-like toxin in the venom of the rabbitfish *Siganus fuscescens*. Toxicon. 2017:140:139–146. 10.1016/j.toxicon.2017.10.015. 29055787

[msad215-B42] Kiriake A , Shiomi K. Some properties and cDNA cloning of proteinaceous toxins from two species of lionfish (*Pterois antennata* and *Pterois volitans*). Toxicon. 2011:58(6-7):494–501. 10.1016/j.toxicon.2011.08.010. 21878347

[msad215-B43] Kiriake A , Suzuki Y, Nagashima Y, Shiomi K. Proteinaceous toxins from three species of scorpaeniform fish (lionfish *Pterois lunulata*, devil stinger *Inimicus japonicus* and waspfish *Hypodytes rubripinnis*): close similarity in properties and primary structures to stonefish toxins. Toxicon. 2013:70:184–193. 10.1016/j.toxicon.2013.04.021. 23665450

[msad215-B44] Kolora SRR , Owens GL, Vazquez JM, Stubbs A, Chatla K, Jainese C, Seeto K, McCrea M, Sandel MW, Vianna J, et al Origins and evolution of extreme life span in Pacific Ocean rockfishes. Science. 2021:374(6569):842–847. 10.1126/science.abg5332. 34762458 PMC8923369

[msad215-B45] Krzywinski M , Schein J, Birol I, Connors J, Gascoyne R, Horsman D, Jones SJ, Marra MA. Circos: an information aesthetic for comparative genomics. Genome Res. 2009:19(9):1639–1645. 10.1101/gr.092759.109. 19541911 PMC2752132

[msad215-B46] Kumar S , Stecher G, Suleski M, Hedges SB. Timetree: a resource for timelines, timetrees, and divergence times. Mol Biol Evol. 2017:34(7):1812–1819. 10.1093/molbev/msx116. 28387841

[msad215-B47] Langmead B , Salzberg SL. Fast gapped-read alignment with Bowtie 2. Nat Methods. 2012:9(4):357–359. 10.1038/nmeth.1923. 22388286 PMC3322381

[msad215-B48] Li B , Dewey CN. RSEM: accurate transcript quantification from RNA-seq data with or without a reference genome. BMC Bioinform. 2011:12(1):323. 10.1186/1471-2105-12-323. PMC3163565 21816040

[msad215-B49] Lima C , Disner GR, Falcão MAP, Seni-Silva AC, Maleski ALA, Souza MM, Reis Tonello MC, Lopes-Ferreira M. The natterin proteins diversity: a review on phylogeny, structure, and immune function. Toxins (Basel). 2021:13(8):538. 10.3390/toxins13080538. 34437409 PMC8402412

[msad215-B50] Liu B , Shi Y, Yuan J, Hu X, Zhang H, Li N, Li Z, Chen Y, Mu D, Fan W. 2013. Estimation of genomic characteristics by analyzing k-mer frequency in de novo genome projects, arXiv. arXiv:1209.3456, preprint: not peer reviewed.

[msad215-B51] Liu Q , Wang X, Xiao Y, Zhao H, Xu S, Wang Y, Wu L, Zhou L, Du T, Lv X, et al Sequencing of the black rockfish chromosomal genome provides insight into sperm storage in the female ovary. DNA Res. 2019:26(6):453–464. 10.1093/dnares/dsz023. 31711192 PMC6993816

[msad215-B52] Love MI , Huber W, Anders S. Moderated estimation of fold change and dispersion for RNA-seq data with DESeq2. Genome Biol. 2014:15(12):550. 10.1186/s13059-014-0550-8. 25516281 PMC4302049

[msad215-B53] Low KSY , Gwee MCE, Yuen R, Gopalakrishnakone P, Khoo HE. Stonustoxin: effects on neuromuscular function in vitro and in vivo. Toxicon. 1994:32(5):573–581. 10.1016/0041-0101(94)90205-4. 8079369

[msad215-B54] Magadum S , Banerjee U, Murugan P, Gangapur D, Ravikesavan R. Gene duplication as a major force in evolution. J Genet. 2013:92(1):155–161. 10.1007/s12041-013-0212-8. 23640422

[msad215-B55] Maillaud C , Hoang-Oppermann T, Hoang-Oppermann V, Rigot H, Girardot S, Nour M. Is stonefish *Synanceia verrucosa* envenomation potentially lethal? Toxicon. 2020:184:78–82. 10.1016/j.toxicon.2020.05.019. 32473254

[msad215-B56] Nguyen LT , Schmidt HA, von Haeseler A, Minh BQ. IQ-TREE: a fast and effective stochastic algorithm for estimating maximum-likelihood phylogenies. Mol Biol Evol. 2015:32(1):268–274. 10.1093/molbev/msu300. 25371430 PMC4271533

[msad215-B57] Poh CH , Yuen R, Khoo HE, Chung M, Gwee M, Gopalakrishnakone P. Purification and partial characterization of stonustoxin (lethal factor) from *Synanceia horrida* venom. Comp Biochem Physiol B. 1991:99(4):793–798. 10.1016/0305-0491(91)90143-2. 1790672

[msad215-B58] Potter S ; Darwin Tree of Life Barcoding collective; Wellcome Sanger Institute Tree of Life programme; Wellcome Sanger Institute Scientific Operations: DNA Pipelines collective; Tree of Life Core Informatics collective; Darwin Tree of Life Consortium. The genome sequence of the long-spined sea scorpion, *Taurulus bubalis* (Euphrasén, 1786). Wellcome Open Res. 2021:6:299. 10.12688/wellcomeopenres.17356.1. 36312458 PMC9587380

[msad215-B59] Prjibelski A , Antipov D, Meleshko D, Lapidus A, Korobeynikov A. Using SPAdes *de novo* assembler. Curr Protoc Bioinform. 2020:70(1):e102. 10.1002/cpbi.102. 32559359

[msad215-B60] Rabosky DL , Chang J, Title PO, Cowman PF, Sallan L, Friedman M, Kaschner K, Garilao C, Near TJ, Coll M, et al An inverse latitudinal gradient in speciation rate for marine fishes. Nature. 2018:559(7714):392–395. 10.1038/s41586-018-0273-1. 29973726

[msad215-B61] Ronquist F , Teslenko M, Mark P, Ayres DL, Darling A, Hohna S, Larget B, Liu L, Suchard MA, Huelsenbeck JP. Mrbayes 3.2: efficient Bayesian phylogenetic inference and model choice across a large model space. Syst Biol. 2012:61(3):539–542. 10.1093/sysbio/sys029. 22357727 PMC3329765

[msad215-B62] Rozewicki J , Li S, Amada KM, Standley DM, Katoh K. MAFFT-DASH: integrated protein sequence and structural alignment. Nucleic Acids Res. 2019:47(W1):W5–W10. 10.1093/nar/gkz342. 31062021 PMC6602451

[msad215-B63] Saggiomo SL , Firth C, Wilson DT, Seymour J, Miles JJ, Wong Y. The geographic distribution, venom components, pathology and treatments of stonefish (*Synanceia* spp.) venom. Mar Drugs. 2021:19(6):302. 10.3390/md19060302. 34073964 PMC8225006

[msad215-B64] Servant N , Varoquaux N, Lajoie BR, Viara E, Chen CJ, Vert JP, Heard E, Dekker J, Barillot E. HiC-Pro: an optimized and flexible pipeline for Hi-C data processing. Genome Biol. 2015:16(1):259. 10.1186/s13059-015-0831-x. 26619908 PMC4665391

[msad215-B65] Simão FA , Waterhouse RM, Ioannidis P, Kriventseva EV, Zdobnov EM. BUSCO: assessing genome assembly and annotation completeness with single-copy orthologs. Bioinformatics. 2015:31(19):3210–3212. 10.1093/bioinformatics/btv351. 26059717

[msad215-B66] Slater GSC , Birney E. Automated generation of heuristics for biological sequence comparison. BMC Bioinform. 2005:6(1):31. 10.1186/1471-2105-6-31. PMC553969 15713233

[msad215-B67] Smith WL , Stern JH, Girard MG, Davis MP. Evolution of venomous cartilaginous and ray-finned fishes. Integr Comp Biol. 2016:56(5):950–961. 10.1093/icb/icw070. 27375272

[msad215-B68] Stanke M , Keller O, Gunduz I, Hayes A, Waack S, Morgenstern B. AUGUSTUS: ab initio prediction of alternative transcripts. Nucleic Acids Res. 2006:34(Web Server):W435–439. 10.1093/nar/gkl200. PMC1538822 16845043

[msad215-B69] Tarailo-Graovac M , Chen N. Using RepeatMasker to identify repetitive elements in genomic sequences. Curr Protoc Bioinform. 2009:Chapter 4:4.10.1–4.10.14. 10.1002/0471250953.bi0410s25. 19274634

[msad215-B70] Trapnell C , Williams BA, Pertea G, Mortazavi A, Kwan G, van Baren MJ, Salzberg SL, Wold BJ, Pachter L. Transcript assembly and quantification by RNA-seq reveals unannotated transcripts and isoform switching during cell differentiation. Nat Biotechnol. 2010:28(5):511–515. 10.1038/nbt.1621. 20436464 PMC3146043

[msad215-B71] Tyanova S , Temu T, Cox J. The MaxQuant computational platform for mass spectrometry-based shotgun proteomics. Nat Protoc. 2016:11(12):2301–2319. 10.1038/nprot.2016.136. 27809316

[msad215-B72] Ueda A , Suzuki M, Honma T, Nagai H, Nagashima Y, Shiomi K. Purification, properties and cDNA cloning of neoverrucotoxin (neoVTX), a hemolytic lethal factor from the stonefish *Synanceia verrucosa* venom. Biochim Biophys Acta. 2006:1760(11):1713–1722. 10.1016/j.bbagen.2006.08.017. 17023116

[msad215-B73] Wang Y , Tang H, DeBarry JD, Tan X, Li J, Wang X, Lee T, Jin H, Marler B, Guo H, et al MCScanx: a toolkit for detection and evolutionary analysis of gene synteny and collinearity. Nucleic Acids Res. 2012:40(7):e49. 10.1093/nar/gkr1293. 22217600 PMC3326336

[msad215-B74] Waterhouse A , Bertoni M, Bienert S, Studer G, Tauriello G, Gumienny R, Heer FT, de Beer TAP, Rempfer C, Bordoli L, et al SWISS-MODEL: homology modelling of protein structures and complexes. Nucleic Acids Res. 2018:46(W1):W296–W303. 10.1093/nar/gky427. 29788355 PMC6030848

[msad215-B75] Wiśniewski JR . Filter aided sample preparation—a tutorial. Anal Chim Acta. 2019:1090:23–30. 10.1016/j.aca.2019.08.032. 31655642

[msad215-B76] Wolf JC , Wolfe MJ. A brief overview of nonneoplastic hepatic toxicity in fish. Toxicol Pathol. 2005:33(1):75–85. 10.1080/01926230590890187. 15805058

[msad215-B77] Xie B , Li X, Lin Z, Ruan Z, Wang M, Liu J, Tong T, Li J, Huang Y, Wen B, et al Prediction of toxin genes from Chinese yellow catfish based on transcriptomic and proteomic sequencing. Int J Mol Sci. 2016:17(4):556. 10.3390/ijms17040556. 27089325 PMC4849012

[msad215-B78] Xie B , Yu H, Kerkkamp H, Wang M, Richardson M, Shi Q. Comparative transcriptome analyses of venom glands from three scorpionfishes. Genomics. 2019:111(3):231–241. 10.1016/j.ygeno.2018.11.012. 30458272

[msad215-B79] Xu Z , Wang H. LTR_FINDER: an efficient tool for the prediction of full-length LTR retrotransposons. Nucleic Acids Res. 2007:35(Web Server):W265–W268. 10.1093/nar/gkm286. 17485477 PMC1933203

[msad215-B80] Yang ZH . PAML 4: phylogenetic analysis by maximum likelihood. Mol Biol Evol. 2007:24(8):1586–1591. 10.1093/molbev/msm088. 17483113

[msad215-B81] Ye C , Hill CM, Wu S, Ruan J, Ma Z. DBG2OLC: efficient assembly of large genomes using long erroneous reads of the third generation sequencing technologies. Sci Rep. 2016:6(1):1. 10.1038/s41598-016-0001-8. 27573208 PMC5004134

[msad215-B82] Yoshinaga-Kiriake A , Nagashima Y, Ishizaki S, Shiomi K. Primary structures and conformations of stonefish toxin-like toxins from three species of rabbitfish, *Siganus puellus*, *Siganus unimaculatus*, and *Siganus virgatus*. Fish Sci. 2020:86(5):889–901. 10.1007/s12562-020-01455-9.

[msad215-B83] Zhang S , Li J, Qin Q, Liu W, Bian C, Yi Y, Wang M, Zhong L, You X, Tang S, et al Whole-genome sequencing of Chinese yellow catfish provides a valuable genetic resource for high-throughput identification of toxin genes. Toxins (Basel). 2018:10(12):488. 10.3390/toxins10120488. 30477130 PMC6316204

[msad215-B84] Ziegman R , Undheim EAB, Baillie G, Jones A, Alewood PF. Investigation of the estuarine stonefish (*Synanceia horrida*) venom composition. J Proteomics. 2019:201:12–26. 10.1016/j.jprot.2019.04.002. 30953730

