# Supplementary material for: A Chromosome-Level Genome Assembly of the Reef Stonefish (Synanceia verrucosa) Provides Novel Insights into Stonustoxin (sntx) Genes
Source: Mol Biol Evol. 2023 Sep 14;40(10):msad215. doi: 10.1093/molbev/msad215 (PMC10566576; doi:10.1093/molbev/msad215)
Supplement: msad215_Supplementary_Data [file msad215_supplementary_data.docx]

**Supplemental Information for:**

**A Chromosome-level Genome Assembly of the Reef Stonefish (*Synanceia verrucosa*) Provides Novel Insights into Stonustoxin (*sntx*) Genes**

Tianle Tang^1†^, Yu Huang^2,3†^, Chao Peng^2,4^, Yanling Liao^1^, Yunyun Lv^5^, Qiong Shi^2,3,5*^ and Bingmiao Gao^1*^

**Supplementary Figures:**


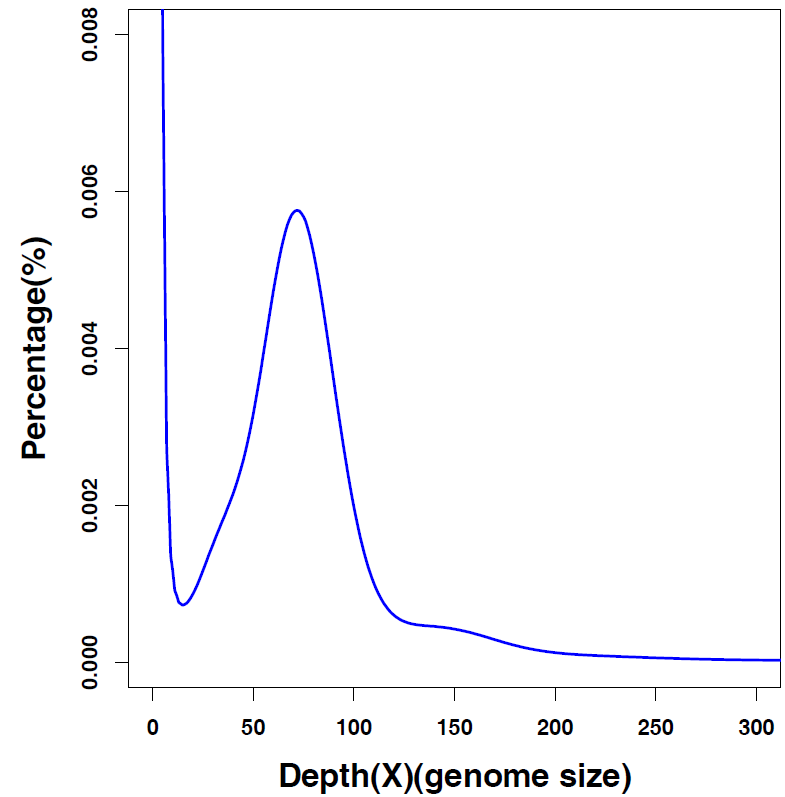


**Figure S1. A K-mer analysis for genome size estimation**. k-mer length was set to be 17 bp. The x-axis represents the sequence depth and the y-axis is the proportion of the frequency at the given depth divided by the total frequency of all depths.


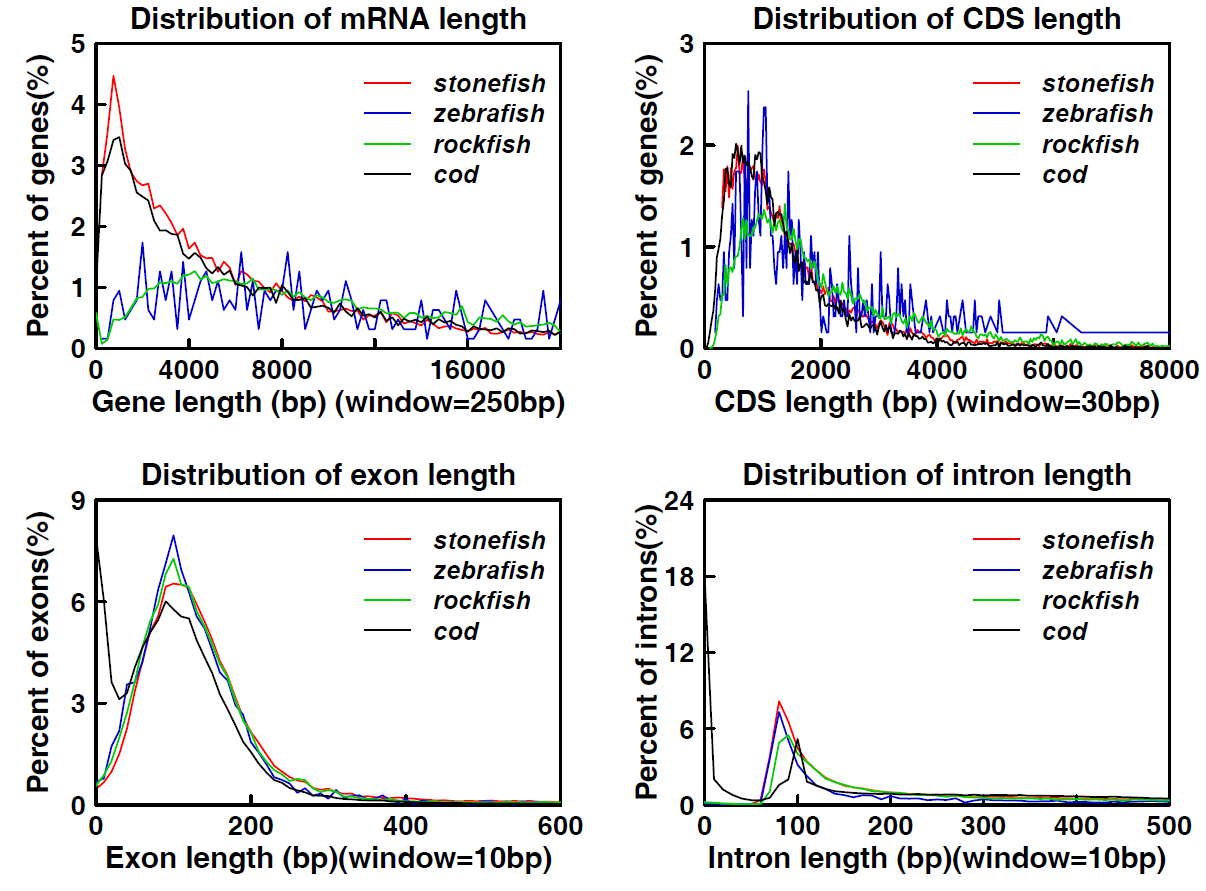


**Figure S2. Comparison of gene structures between the reef stonefish and other examined teleosts.** Red, blue, green and black lines refer to the reef stonefish, zebrafish, honeycomb rockfish, and Atlantic cod, respectively.


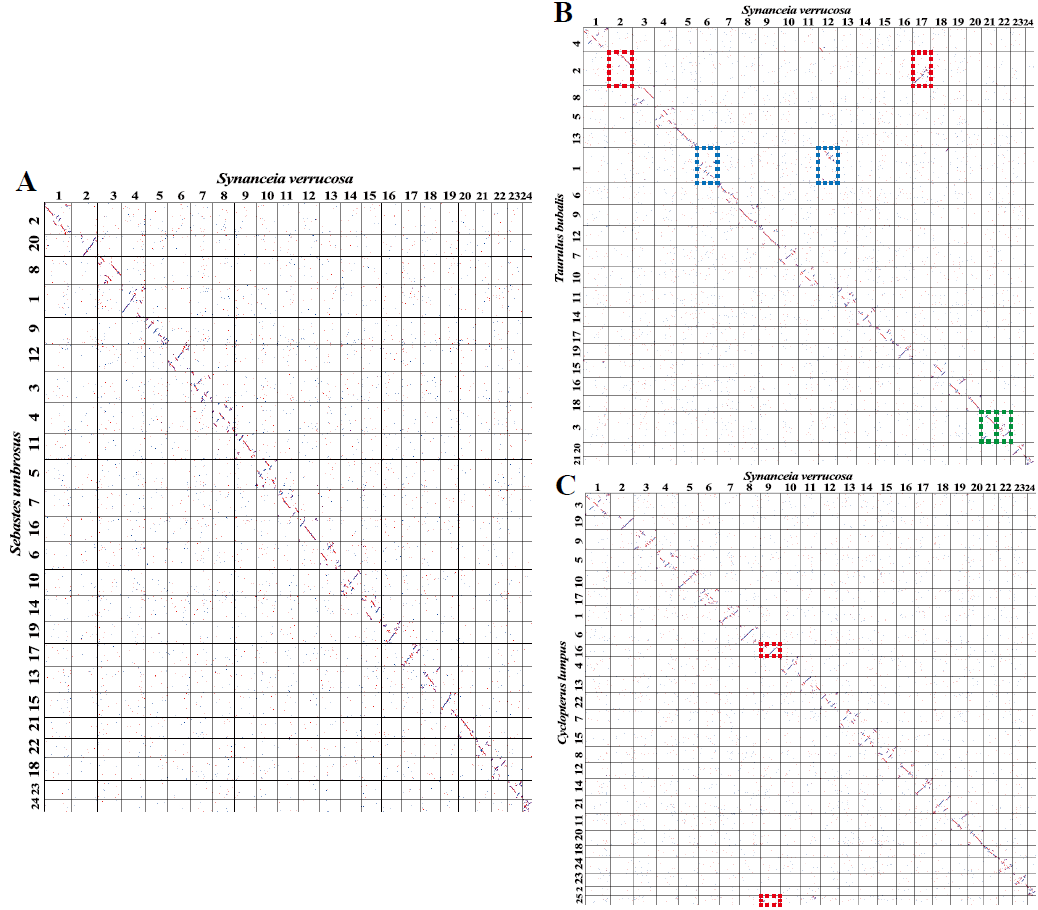


**Figure S3. Chromosomal comparisons between the reef stonefish and other Scorpaeniformes species.** (**A**) Chromosome synteny of the reef stonefish and honeycomb rockfish (2n=48). (**B**) Chromosome synteny of the reef stonefish and long-spined sea scorpion (2n=42). Red, blue and green boxes denote the chromosomes showing one-to-two relationship. For example, Chr2 in the sea scorpion corresponds to Chr2 & 17 of the stonefish (two red boxes). (**C**) Chromosome synteny of the reef stonefish and common lumpfish (2n=50). The two red boxes denoting Chr16 & 25 of the lumpfish correspond to only one chromosome (Chr9) of the stonefish.


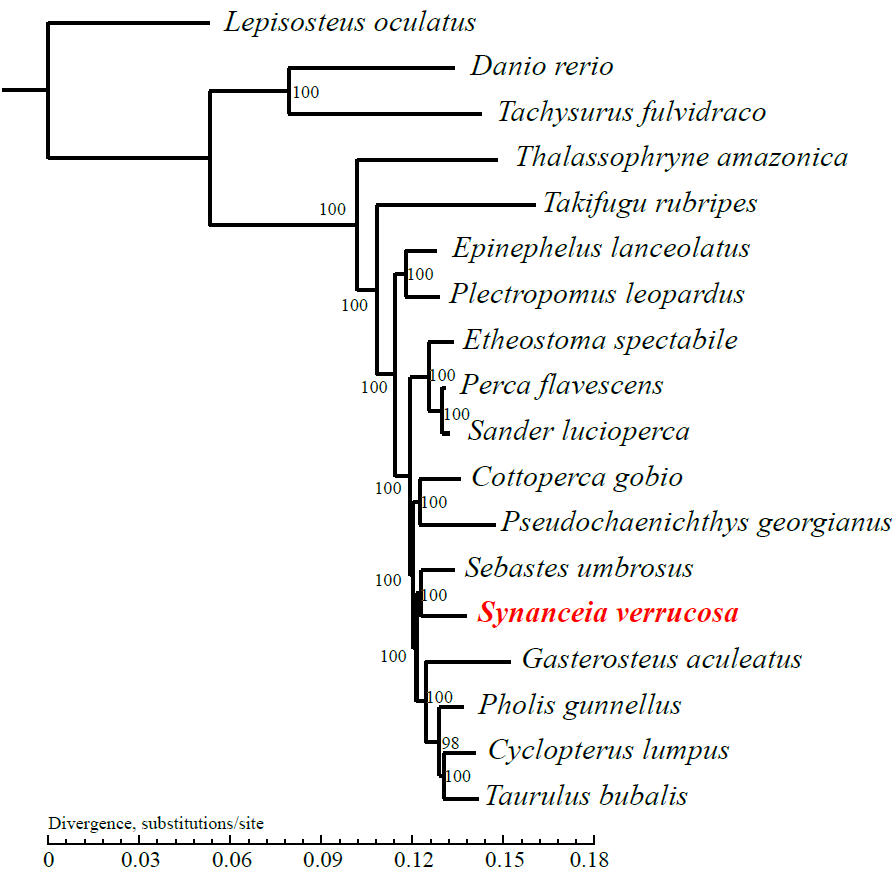


**Figure S4. Species tree constructed by PhyML based on the Maximum Likelihood method.** Bootstrap values are presented at each node. The reef stonefish is highlighted in red.


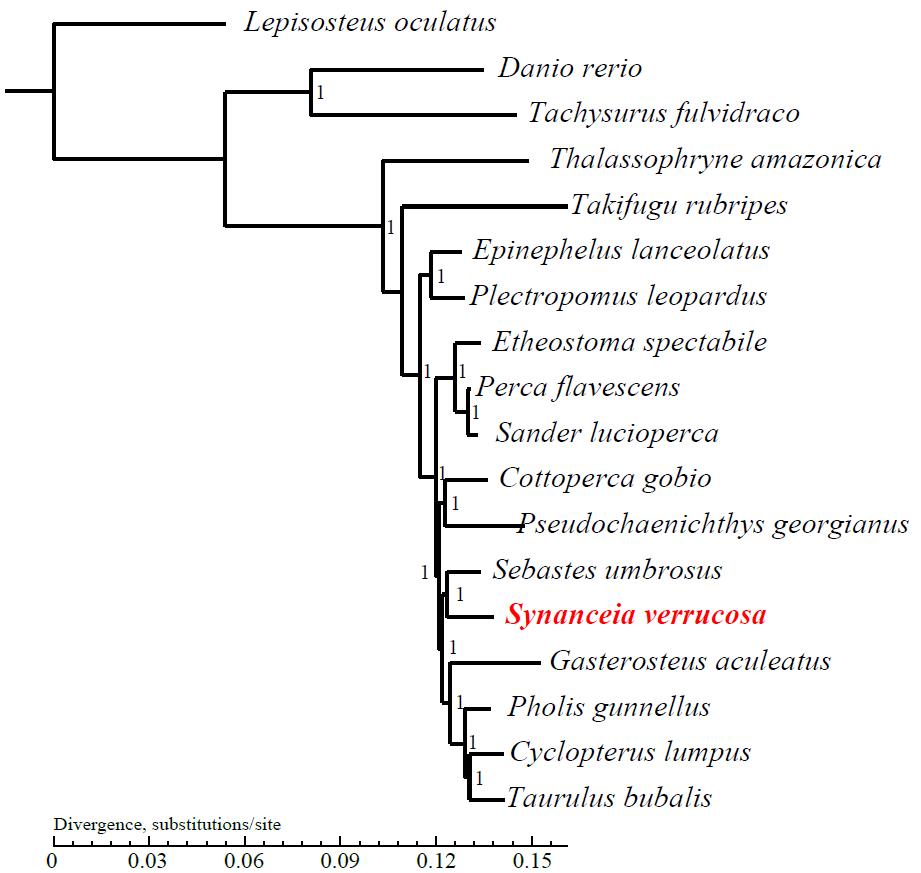


**Figure S5. Species tree constructed by MrBayes based on the Bayesian inference method.** Node supported values are presented at each node. The reef stonefish is highlighted in red.


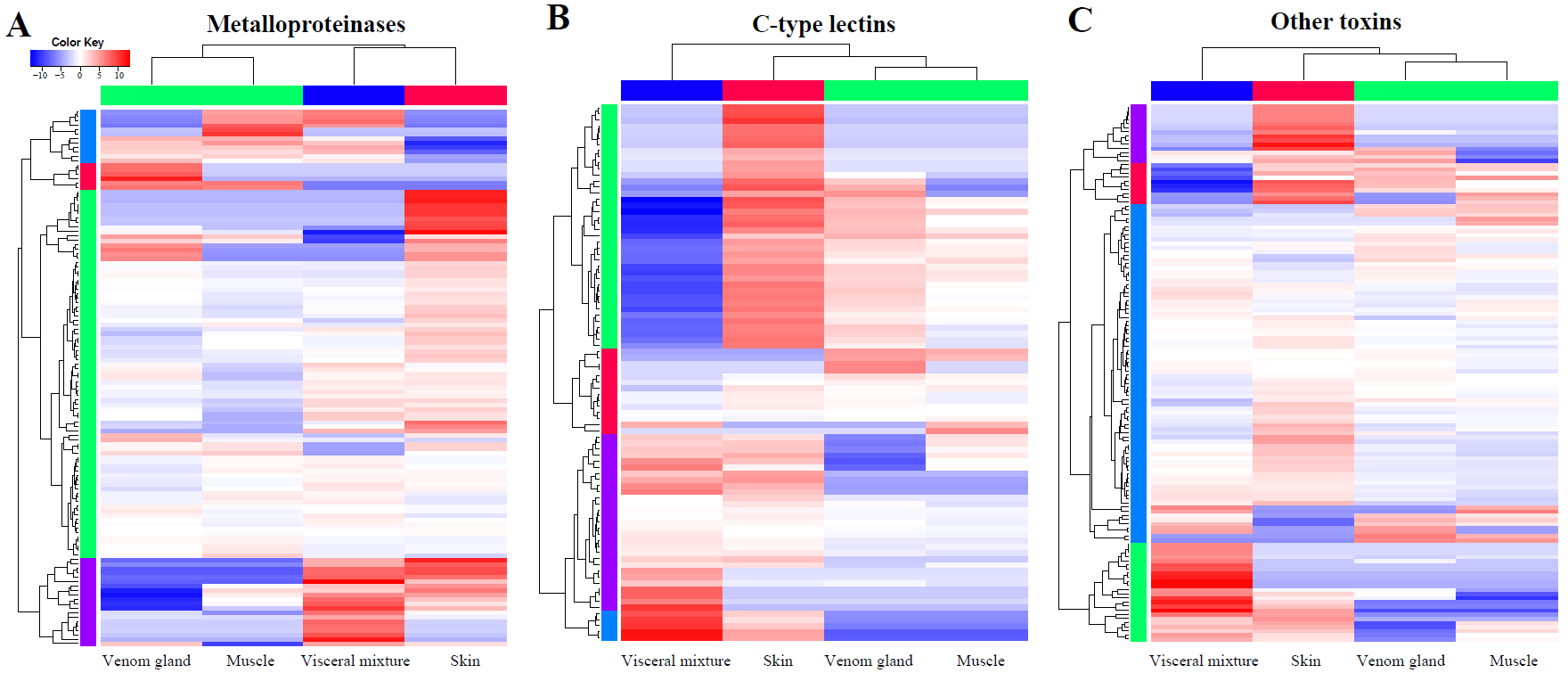


**Figure S6. Transcription levels of toxin gene in the venom gland, muscle, skin, and visceral mixture. (A)** Heatmap of gene transcription with hierarchical clustering for metalloproteinase genes. **(B)** Heatmap of gene transcription with hierarchical clustering for the C-type lectin genes. **(C)** Heatmap of gene transcription with hierarchical clustering for the other types of toxin genes.


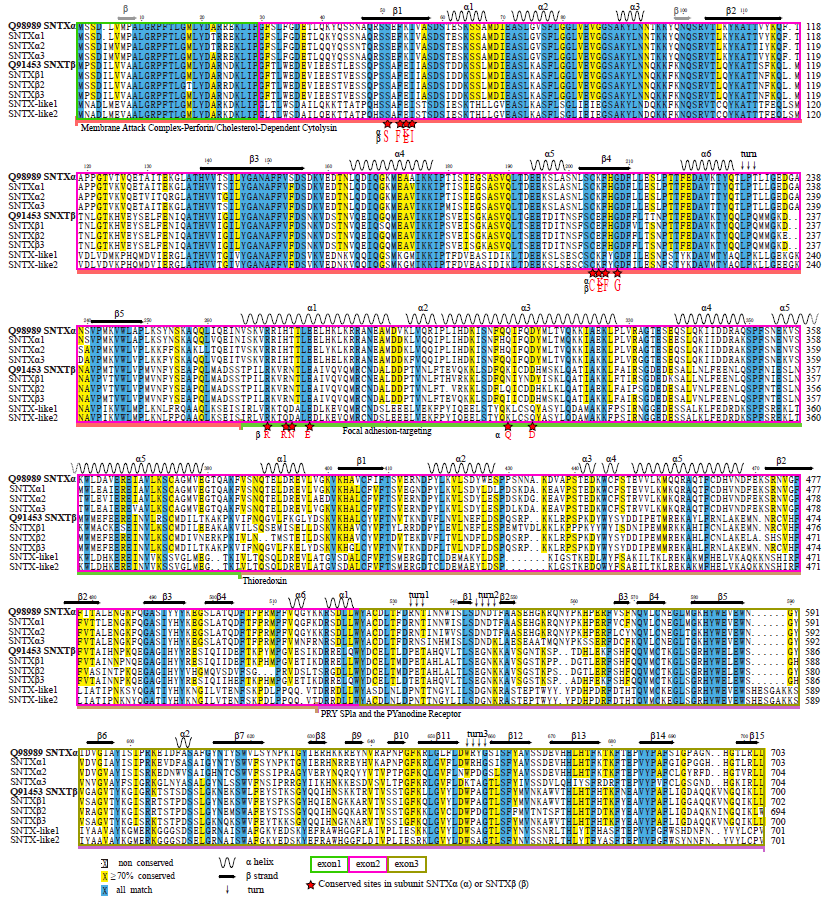


**Figure S7. Protein sequence alignment of the eight SNTXs clustered within a 50-kb region on the Chr2.** Secondary structures (α helix, β strand, and turn) are labeled above the sequences, and the four conserved domains are marked below. Red stars refer to the conserved sites. Two reference sequences with accession numbers are also included.

**
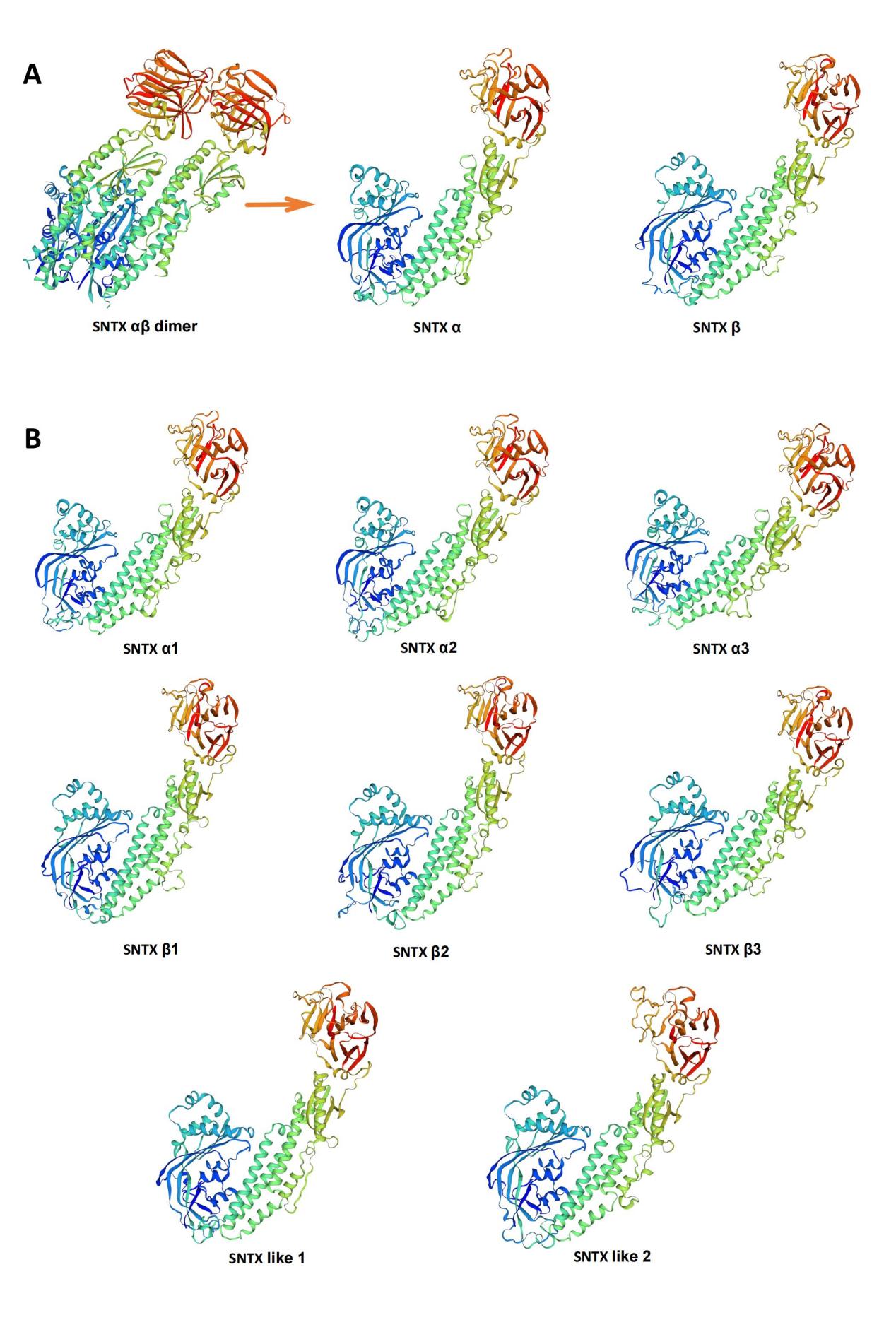
**

**Figure S8. 3D structures of various SNTXs. (A)** The reference 3D structure of the Stonustoxin (PDB: 4WVM) including SNTX αβ dimer, α and β subunits from stonefish *Synanceia horrida.* **(B)** The predicted 3D structures of eight SNTXs including three SNTX-α subunits, three SNTX-β subunits, and two SNTX-like proteins were built using the SWISS-Model server.

**Supplementary Tables:**

**Table S1:** Statistics of the Illumina paired-end short reads for the reef stonefish.

| **Platform** | **Reads** | **Insert size (bp)** | **Length (bp)** | **Read pairs** | **Bases** |
| --- | --- | --- | --- | --- | --- |
| Illumina | Raw | 300 | 150_150 | 190,141,313 | 57,042,393,900 |
|  | Clean | 300 | 140_140 | 180,063,823 | 50,417,870,440 |

**Table S2:** Statistics of the Nanopore long reads for the reef stonefish.

| **Platform** | **Reads** | **Read No.** | **Base No.** | **Average Length (bp)** | **Max Length (bp)** |
| --- | --- | --- | --- | --- | --- |
| Oxford Nanopore | Raw | 5,889,814 | 128,197,477,346 | 22,958.55 | 384,572 |
|  | Clean | 4,804,911 | 110,313,790,401 | 22,958.60 | 384,572 |

**Table S3:** Primary genome assembly of the reef stonefish using the short and long reads.

| **Contig** | **Length (bp)** | | **Number** |
| --- | --- | --- | --- |
| Max length | 25,393,742 | |  |
| N10 | 22,746,671 | | 3 |
| N20 | 22,202,722 | | 6 |
| N30 | 18,164,148 | | 10 |
| N40 | 16,005,273 | | 14 |
| N50 | 11,969,388 | | 19 |
| N60 | 8,916,596 | | 25 |
| N70 | 6,234,141 | | 34 |
| N80 | 2,404,246 | | 52 |
| N90 | 152,878 | | 176 |
| Total length | 689,521,312 | | 1370 |
| Number of contigs ≥ 100 bp | | 2,799 | |
| Number of contigs ≥ 2 kb | | 2,794 | |
| GC Ratio | | 40.7% | |

**Table S4.** Summary of the Hi-C sequencing and mapping for the reef stonefish.

| **Item** | **Number** | **Percentage (%)** |
| --- | --- | --- |
| Sequenced read bases | 131,138,547,900 | 100 |
| Sequenced read pairs (PE150) | 437,128,493 | 100 |
| Unmapped read pairs | 6,000,514 | 1.37 |
| Unique paired alignments | 254,658,517 | 58.26 |
| Multiple paired alignments | 109,151,530 | 24.97 |
| Pairs with singleton | 67,317,932 | 15.40 |
| Reported pairs | 254,658,517 | 58.26 |
| Valid interaction | 202,292,743 | 46.28 |
| Valid interaction without duplicates | 192,116,350 | 43.95 |

**Table S5.** Statistics of the final chromosome-level genome assembly of the reef stonefish.

| **Item** | | **Scaffold** | | **Contig** | |
| --- | --- | --- | --- | --- | --- |
|  |  | **Length (bp)** | **Number** | **Length (bp)** | **Number** |
| Max length | | 35,828,527 |  | 25,479,283 |  |
| N10 | | 33,428,630 | 2 | 22,369,487 | 3 |
| N20 | | 28,798,446 | 5 | 21,717,896 | 6 |
| N30 | | 28,476,703 | 7 | 16,536,915 | 10 |
| N40 | | 27,775,071 | 10 | 16,037,899 | 14 |
| N50 | | 27,113,652 | 12 | 12,009,665 | 19 |
| N60 | | 26,375,978 | 15 | 8,467,849 | 26 |
| N70 | | 25,215,824 | 17 | 6,133,395 | 35 |
| N80 | | 20,916,278 | 20 | 2,302,085 | 56 |
| N90 | | 11,346,221 | 24 | 96,253 | 229 |
| Total length | | 689,736,203 | | 689,343,153 | |
| Number of contigs ≥ 100 bp | 1,437 | | 2,230 | |  |
| Number of contigs ≥ 2 kb | 1,390 | | 2,179 | |  |
| GC ratio | 40.8% | | 40.9% | |  |

**Table S6.** Summary of the 24 assembled pseudo-chromosomes of the reef stonefish.

| **Chromosome** | **Length without gap (bp)** | **Total length (bp)** | **GC content** |
| --- | --- | --- | --- |
| Chr1 | 35,820,527 | 35,828,527 | 40.84% |
| Chr2 | 33,272,630 | 33,428,630 | 41.09% |
| Chr3 | 31,612,000 | 31,616,500 | 41.08% |
| Chr4 | 31,028,154 | 31,034,154 | 40.95% |
| Chr5 | 28,795,946 | 28,798,446 | 40.78% |
| Chr6 | 28,574,054 | 28,583,054 | 41.01% |
| Chr7 | 28,475,203 | 28,476,703 | 40.55% |
| Chr8 | 28,443,110 | 28,446,611 | 40.85% |
| Chr9 | 28,187,303 | 28,192,803 | 40.99% |
| Chr10 | 27,770,071 | 27,775,071 | 40.70% |
| Chr11 | 27,366,870 | 27,370,871 | 40.77% |
| Chr12 | 27,087,652 | 27,113,652 | 41.04% |
| Chr13 | 26,684,341 | 26,686,841 | 40.70% |
| Chr14 | 26,583,681 | 26,591,681 | 40.93% |
| Chr15 | 26,369,478 | 26,375,978 | 41.36% |
| Chr16 | 25,425,510 | 25,449,010 | 40.32% |
| Chr17 | 25,207,324 | 25,215,824 | 40.52% |
| Chr18 | 24,676,257 | 24,683,257 | 40.61% |
| Chr19 | 23,976,742 | 23,985,242 | 40.90% |
| Chr20 | 20,913,278 | 20,916,278 | 40.77% |
| Chr21 | 20,519,488 | 20,537,488 | 40.45% |
| Chr22 | 20,406,999 | 20,408,999 | 40.73% |
| Chr23 | 19,955,000 | 19,956,500 | 40.74% |
| Chr24 | 11,340,721 | 11,346,221 | 41.98% |

**Table S7.** BUSCO completeness assessment of the reef stonefish genome.

| **BUSCO category** | **Vertebrata_odb9** |
| --- | --- |
| Complete BUSCOs | 4,482 (97.8%) |
| Complete and single-copy BUSCOs | 4,348 (94.9%) |
| Complete and duplicated BUSCOs | 134 (2.9%) |
| Fragmented BUSCOs | 36 (0.8%) |
| Missing BUSCOs | 66 (1.4%) |
| Total BUSCO groups searched | 4,584 |

**Table S8.** Statistics of repetitive sequences in the reef stonefish genome.

| **Type** | **Repbase TEs** | | **TE Protiens** | | **De novo** | | **Combined TEs** | | |  |
| --- | --- | --- | --- | --- | --- | --- | --- | --- | --- | --- |
|  | **Length**  **(bp)** | **Percent**  **(%)** | **Length**  **(bp)** | **Percent**  **(%)** | **Length**  **(bp)** | **Percent**  **(%)** | | **Length**  **(bp)** | **Percent**  **(%)** | |
| DNA | 27791435 | 4.02 | 8894256 | 1.29 | 104427547 | 15.10 | | 116401124 | 16.83 | |
| LINE | 19200901 | 2.78 | 15459127 | 2.24 | 56935922 | 8.23 | | 66878947 | 9.67 | |
| SINE | 8699707 | 1.26 | 0 | 0.00 | 11186089 | 1.62 | | 18383632 | 2.66 | |
| LTR | 12766019 | 1.85 | 8475326 | 1.23 | 36953896 | 5.34 | | 43654874 | 6.31 | |
| Other | 10081 | 0.00 | 0 | 0.00 | 0 | 0.00 | | 10081 | 0.00 | |
| Unknown | 0 | 0.00 | 0 | 0.00 | 9148176 | 1.32 | | 9148176 | 1.32 | |
| Total | 62722140 | 9.07 | 32778051 | 4.74 | 197847813 | 28.61 | | 211542438 | 30.59 | |

**Table S9.** Fish genomes and accession numbers used for gene annotation and phylogeny analysis.

| **Scientific Name** | **Order** | **Family** | **NCBI Accession** |
| --- | --- | --- | --- |
| *Synanceia verrucose*^†^ | Perciformes | Synanceiidae | Our present study |
| *Sebastes umbrosus*^‡^ | Perciformes | Sebastidae | GCA_015220745.1 |
| *Pseudochaenichthys georgianus*^‡^ | Perciformes | Channichthyidae | GCA_902827115.1 |
| *Cottoperca gobio* | Perciformes | Bovichtidae | GCA_900634415.1 |
| *Epinephelus lanceolatus* | Perciformes | Serranidae | GCA_005281545.1 |
| *Plectropomus leopardus* | Perciformes | Serranidae | GCA_008729295.2 |
| *Gasterosteus aculeatus*^‡^ | Perciformes | Gasterosteidae | GCA_016920845.1 |
| *Cyclopterus lumpus*^‡^ | Perciformes | Cyclopteridae | GCA_009769545.1 |
| *Pholis gunnellus* | Perciformes | Pholidae | GCA_910591455.2 |
| *Taurulus bubalis* | Perciformes | Cottidae | GCA_910589615.1 |
| *Etheostoma spectabile* | Perciformes | Percidae | GCA_008692095.1 |
| *Sander lucioperca*^‡^ | Perciformes | Percidae | GCA_008315115.2 |
| *Perca flavescens*^‡^ | Perciformes | Percidae | GCA_004354835.1 |
| *Lepisosteus oculatus* | Lepisosteiformes | Lepisosteidae | GCA_000242695.1 |
| *Danio rerio*^‡^ | Cypriniformes | Cyprinidae | GCA_000002035.4 |
| *Takifugu rubripes*^‡^ | Tetraodontiformes | Tetraodontidae | GCA_901000725.2 |
| *Tachysurus fulvidraco*^†^ | Siluriformes | Bagridae | GCA_022655615.1 |
| *Thalassophryne amazonica*^†^ | Batrachoidiformes | Batrachoididae | GCA_902500255.1 |

Note: Known venomous species are marked with “^†^”. Species used for gene annotation are marked with “^‡^”.

**Table S10.** Gene functional annotation against various protein databases.

| **Values** | **Total** | **Swissprot** | **KEGG** | **TrEMBL** | **Interpro** | **Overall** |
| --- | --- | --- | --- | --- | --- | --- |
| Number | 24,050 | 20,627 | 21,335 | 23,765 | 22,063 | 23,779 |
| Percentage | 100% | 85.77% | 88.71% | 98.81% | 91.74% | 98.87% |

**Table S11.** Gene family clusters in 18 examined fishes including the reef stonefish.

| **Species** | **Gene**  **number** | **Genes in families** | **Unflustered genes** | **Family number** | **Unique families** | **Genes /family** |
| --- | --- | --- | --- | --- | --- | --- |
| *Cottoperca gobio* | 21,272 | 20,903 | 369 | 15,414 | 29 | 1.36 |
| *Cyclopterus lumpus* | 21,480 | 21,235 | 245 | 15,836 | 23 | 1.34 |
| *Danio rerio* | 26,458 | 24,473 | 1985 | 15,197 | 192 | 1.61 |
| *Epinephelus lanceolatus* | 24,165 | 23,433 | 732 | 16,860 | 65 | 1.39 |
| *Etheostoma spectabile* | 22,328 | 21,480 | 848 | 15,644 | 40 | 1.37 |
| *Gasterosteus aculeatus* | 20,787 | 19,615 | 1,172 | 14,264 | 34 | 1.38 |
| *Lepisosteus oculatus* | 18,341 | 16,974 | 1,367 | 13,501 | 99 | 1.26 |
| *Perca flavescens* | 23,736 | 23,000 | 736 | 16,524 | 37 | 1.39 |
| *Pholis gunnellus* | 23,096 | 22,065 | 1,031 | 16,015 | 96 | 1.38 |
| *Plectropomus leopardus* | 25,789 | 22,296 | 3,493 | 16,389 | 70 | 1.36 |
| *Pseudochaenichthys georgianus* | 23,287 | 22,378 | 909 | 15,710 | 76 | 1.42 |
| *Sander lucioperca* | 24,714 | 24,072 | 642 | 17,045 | 56 | 1.41 |
| *Sebastes umbrosus* | 23,881 | 23,472 | 409 | 16,645 | 56 | 1.41 |
| *Synanceia verrucose* | 24,050 | 22,288 | 1,762 | 14,866 | 210 | 1.5 |
| *Tachysurus fulvidraco* | 23,556 | 22,347 | 1,209 | 15,052 | 179 | 1.48 |
| *Takifugu rubripes* | 18,518 | 17,908 | 610 | 13,130 | 42 | 1.36 |
| *Taurulus bubalis* | 25,414 | 23,470 | 1,944 | 16,075 | 278 | 1.46 |
| *Thalassophryne amazonica* | 22,351 | 21,222 | 1,129 | 15,376 | 97 | 1.38 |

**Table S12.** Sequence alignments for the phylogeny of 18 fish species and the stonustoxins.

| **Link/DOI** | **Description** |
| --- | --- |
| https://doi.org/10.6084/m9.figshare.23295032 | Alignments for construction of the species tree |
| https://doi.org/10.6084/m9.figshare.23295089 | Alignments of SNTXs from all studied fish |
| https://doi.org/10.6084/m9.figshare.23295134 | Alignments of SNTXs on Chr02 |

**Table S13.** Transcriptome sequencing of various tissues of the reef stonefish.

| **Full-length Transcriptome Sequencing (Iso-Seq)** | | | | | | | |  |
| --- | --- | --- | --- | --- | --- | --- | --- | --- |
| **Tissue type** | **Reads number** | **Mean Length**  **(bp)** | **Full-length reads** | | **Total isoform** | **Novel isoform** | | |
| Venom gland | 20,529,437 | 2,708 | 566,784 | | 158,158 | 29,734 | | |
| **Short-read Transcriptome Sequencing (RNA-seq)** | | | | | | | |  |
| **Tissue type** | **Raw bases**  **(bp)** | **Clean bases**  **(bp)** | | **Mapping ratio** | **Total**  **transcripts** | | **Novel transcript** | |
| Venom gland | 8,987,349,300 | 8,603,370,900 | | 85.30% | 25,327 | | 16,071 | |
| Muscle | 16,059,856,800 | 15,373,807,200 | | 87.67% | 26,685 | | 16,333 | |
| Skin | 11,159,193,000 | 10,673,968,500 | | 85.56% | 28,294 | | 16,514 | |
| Visceral mixture | 14,282,169,000 | 13,734,432,000 | | 91.02% | 26,934 | | 16,005 | |

**Table S14.** Top 30 peptides with the highest density in the venom proteome data.

| **No.** | **Sequence** | **Protein** | **Score** | **Intensity**  **(×10^6^)** | **Protein Annotation** |
| --- | --- | --- | --- | --- | --- |
| 1 | IVASDSTESK | ST_0001123 | 144.09 | 60,702 | Stonustoxin |
| 2 | LLTQEITVSK | ST_0001123 | 168.25 | 41,519 | Stonustoxin |
| 3 | VQETVITQR | ST_0001123 | 223.55 | 41,438 | Stonustoxin |
| 4 | LQATIAK | ST_0001121; ST_0001124 | 132.09 | 31,720 | Stonustoxin |
| 5 | SCMDILTK | ST_0001124 | 157.33 | 31,028 | Stonustoxin |
| 6 | KIPSVEISGK | ST_0001121; ST_0001124 | 112.83 | 29,402 | Stonustoxin |
| 7 | AGTESEQSLQK | ST_0001123 | 309.63 | 29,310 | Stonustoxin |
| 8 | SCAGMVEGTQAK | ST_0001123 | 215.06 | 28,370 | Stonustoxin |
| 9 | LVQSEEAASRRR | ST_0005725 | 73.985 | 27,749 | Sickle tail protein homolog |
| 10 | SLASNLSCK | ST_0001123 | 168.27 | 27,529 | Stonustoxin |
| 11 | HGKRMGYTFDNK | ST_0001007 | 76.827 | 27,384 | Dynein heavy chain 9 |
| 12 | QLMTNLGTK | ST_0001121; ST_0001124 | 175.51 | 25,540 | Stonustoxin |
| 13 | YESCGPGWTQVPENR | ST_0010703 | 322.23 | 22,218 | Distal membrane-arm assembly complex protein 2 |
| 14 | VLSDYLESPDSKDGK | ST_0001123 | 308.6 | 21,330 | Stonustoxin |
| 15 | QFTAPPGTVK | ST_0001123 | 124.74 | 21,201 | Stonustoxin |
| 16 | TYQQLPQMMGK | ST_0001121; ST_0001124 | 162.59 | 20,904 | Stonustoxin |
| 17 | MPFVQGYK | ST_0001123 | 153.61 | 20,617 | Stonustoxin |
| 18 | IAEKLPLVR | ST_0001123 | 188.76 | 20,164 | Stonustoxin |
| 19 | AQTFCDHVNDFEK | ST_0001123 | 291.93 | 19,500 | Stonustoxin |
| 20 | AVSGSTK | ST_0001121 | 114.19 | 18,955 | Stonustoxin |
| 21 | EGSLATQDFTFPR | ST_0001123 | 250.76 | 18,332 | Stonustoxin |
| 22 | FVSNQTELDREVLAEDVK | ST_0001123 | 248.06 | 17,651 | Stonustoxin |
| 23 | LSDFQIICDDHMSK | ST_0001124 | 227.93 | 16,207 | Stonustoxin |
| 24 | VPAHAVVTR | ST_0005269 | 153.78 | 15,295 | Serotransferrin |
| 25 | ANEAMDDKLVQQIPLIHDK | ST_0001123 | 461.43 | 14,248 | Stonustoxin |
| 26 | EMTWSDAEK | ST_0002096 | 136.27 | 13,393 | Type-2 ice-structuring protein |
| 27 | ELTWTGAEK | ST_0002098 | 141.52 | 13,282 | Type-2 ice-structuring protein |
| 28 | EITALAPSTMK | ST_0000473; ST_0019565  ST_0011023; ST_0001073  ST_0005948; ST_0011206 | 154.56 | 12,896 | Actin |
| 29 | QEGAGIHYYR | ST_0001124 | 332.49 | 12,524 | Stonustoxin |
| 30 | MPTDHPMVK | ST_0010469 | 137.90 | 11,917 | Alpha-2-macroglobulin |

**Table S15.** Sequence identity matrix of the stonustoxin genes on the Chr2 of the reef stonefish.

| **ID** | **Q98989** | **α1** | **α2** | **α3** | **Q91453** | **β1** | **β2** | **β3** | **like1** | **like2** |
| --- | --- | --- | --- | --- | --- | --- | --- | --- | --- | --- |
| **Q98989** | **-** | 92.70% | 86.50% | 83.50% | 47.80% | 48.50% | 47.10% | 48.10% | 45.70% | 45.90% |
| **α1** | 92.70% | - | 89.60% | 86.20% | 48.80% | 48.30% | 48.00% | 49.10% | 46.30% | 46.40% |
| **α2** | 86.50% | 89.60% | - | 86.60% | 48.50% | 48.90% | 48.00% | 49.10% | 46.60% | 46.70% |
| **α3** | 83.50% | 86.20% | 86.60% | - | 48.50% | 48.70% | 47.40% | 48.70% | 46.40% | 46.60% |
| **Q91453** | 47.80% | 48.80% | 48.50% | 48.50% | - | 86.60% | 84.10% | 94.50% | 46.20% | 46.30% |
| **β1** | 48.50% | 48.30% | 48.90% | 48.70% | 86.60% | - | 83.30% | 88.40% | 45.70% | 46.20% |
| **β2** | 47.10% | 48.00% | 48.00% | 47.40% | 84.10% | 83.30% | - | 85.20% | 44.90% | 45.20% |
| **β3** | 48.10% | 49.10% | 49.10% | 48.70% | 94.50% | 88.40% | 85.20% | - | 46.20% | 46.20% |
| **like1** | 45.70% | 46.30% | 46.60% | 46.40% | 46.20% | 45.70% | 44.90% | 46.20% | - | 97.80% |
| **like2** | 45.90% | 46.40% | 46.70% | 46.60% | 46.30% | 46.20% | 45.20% | 46.20% | 97.80% | - |
